# An IKK/NF-κB Activation/p53 Deletion Sequence Drives Liver Carcinogenesis and Tumor Differentiation

**DOI:** 10.3390/cancers11101410

**Published:** 2019-09-21

**Authors:** Michael Svinarenko, Sarah-Fee Katz, Umesh Tharehalli, Medhanie A. Mulaw, Harald J. Maier, Yoshiaki Sunami, Sarah K. Fischer, Yuexin Chen, Sabine Heurich, Lena Erkert, Andrea Tannapfel, Thomas Wirth, Reinhold Schirmbeck, Thomas Seufferlein, André Lechel

**Affiliations:** 1 Department of Internal Medicine I, University Hospital Ulm, 89081 Ulm, Germany; michael.svinarenko@uni-ulm.de (M.S.); sarah-fee.katz@web.de (S.-F.K.); umesh.tharehalli@uni-ulm.de (U.T.); maierhj@gmail.com (H.J.M.); sarah.k.f.91@googlemail.com (S.K.F.); yuexin.chen@tum.de (Y.C.); sabine.heurich@uni-ulm.de (S.H.); lena.erkert@t-online.de (L.E.); Reinhold.Schirmbeck@uniklinik-ulm.de (R.S.); Thomas.Seufferlein@uniklinik-ulm.de (T.S.); 2 Institute of Experimental Cancer Research, Comprehensive Cancer Center Ulm, University Hospital Ulm, 89081 Ulm, Germany; medhanie.mulaw@uni-ulm.de; 3 Institute of Physiological Chemistry, Ulm University, 89081 Ulm, Germany; thomas.wirth@uni-ulm.de; 4 Department of Visceral, Vascular and Endocrine Surgery, Halle University Hospital, Martin-Luther University Halle-Wittenberg, 06120 Halle, Germany; yoshiaki.sunami@uk-halle.de; 5 Center for Translational Cancer Research (TranslaTUM), Technical University Munich, 81675 Munich, Germany; 6 Department of Internal Medicine II, Klinikum rechts der Isar, Technical University Munich, 81675 Munich, Germany; 7 Institute of Pathology, Ruhr University Bochum, 44789 Bochum, Germany; andrea.tannapfel@pathologie-bochum.de

**Keywords:** inflammation, hepatocellular carcinoma, intrahepatic cholangiocarcinoma, ectopic lymphoid structures, progenitor cells

## Abstract

Background: Most liver tumors arise on the basis of chronic liver diseases that trigger inflammatory responses. Besides inflammation, subsequent defects in the p53-signaling pathway frequently occurs in liver cancer. In this study, we analyzed the consequences of inflammation and p53 loss in liver carcinogenesis. Methods: We used inducible liver-specific transgenic mouse strains to analyze the consequences of NF-κB/p65 activation mimicking chronic inflammation and subsequent p53 loss. Results: *Ikk2^ca^* driven NF-κB/p65 activation in mice results in liver fibrosis, the formation of ectopic lymphoid structures and carcinogenesis independent of p53 expression. Subsequent deletion of *Trp53* led to an increased tumor formation, metastasis and a shift in tumor differentiation towards intrahepatic cholangiocarcinoma. In addition, loss of *Trp53* in an inflammatory liver resulted in elevated chromosomal instability and indicated a distinct aberration pattern. Conclusions: In conclusion, activation of NF-κB/p65 mimicking chronic inflammation provokes the formation of liver carcinoma. Collateral disruption of *Trp53* supports tumor progression and influences tumor differentiation and heterogeneity.

## 1. Introduction

Liver carcinoma is one of the most common cancers and the second leading cause of cancer-related death worldwide [1]. About 80% of all liver cancers arise as a consequence of chronic liver disease (CLD) and occur in an intense inflammatory environment. Indeed, chronic inflammation and the associated wound-healing response are strongly linked with the development of fibrosis, followed by cirrhosis, and hepatocellular carcinoma (HCC) [2]. Consequently, about 80% of all HCCs are preceded by liver fibrosis and cirrhosis [3]. A close interplay between inflammation and carcinogenesis was suggested by several transgenic mouse models [4,5,6,7,8]. However, the precise mechanisms mediating inflammation-induced carcinogenesis, particularly in the liver, remain to be defined. NF-κB is a key player in the regulation of inflammation and is activated in the majority of CLD, including alcoholic and nonalcoholic fatty liver disease, viral hepatitis and biliary liver diseases [9,10,11,12,13]. NF-κB plays a central role in the liver. In general activation of NF-κB in parenchymal and non-parenchymal cells promotes inflammation, liver fibrosis, and hepatocarcinogenesis [14,15,16,17,18]. Interestingly, inhibition of NF-κB activation in parenchymal cells results in promotion or inhibition of hepatocarcinogenesis, depending on the experimental setting [17,18,19]. Subsequent to the inflammatory response, 12–48% of liver cancers exhibit mutations in the *TP53* gene or a disruption of the p53 signaling pathway [20] leading to poorly differentiated tumors and distinct gene expression profiles, which are linked to stemness [21]. We have previously shown that *Trp53* deletion as a single genetic lesion results in the formation of bilineal differentiated liver carcinomas [22].

The precise contribution of chronic inflammation and a subsequent loss of p53 to tumor initiation, tumor progress, and tumor differentiation in the liver are not well described. Here, we analyze the consequence of NF-κB activation mimicking chronic inflammation and *Trp53* deletion in liver carcinogenesis and tumor differentiation.

## 2. Results

### 2.1. Generation of Inducible Ikk2^ca^ Trp53^−/−^ Mice to Study the Consequences of Inflammation and p53 Loss

**Figure 1:**
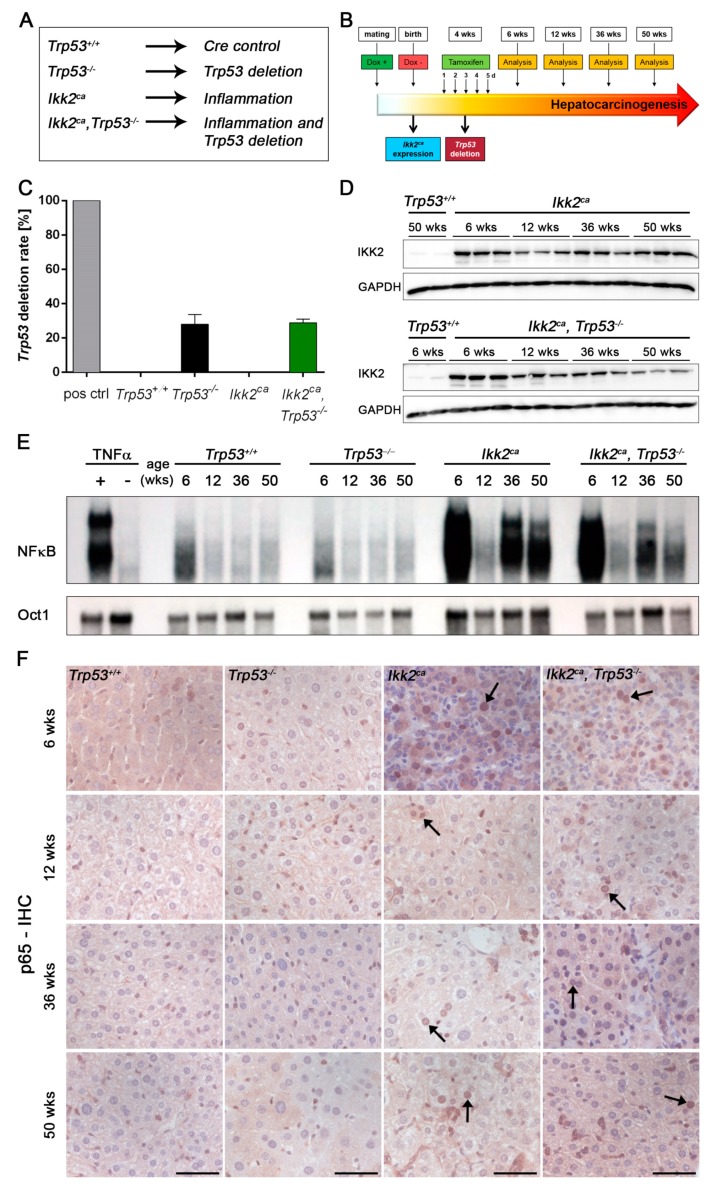
Mouse model of inducible NF-κB activation and p53 loss. (**A**) Experimental genetic cohorts for the study of the sequence of induced inflammation and p53 loss. (**B**) Experimental study design of liver-specific induction of *Ikk2^ca^* overexpression (Tet-off system) and tamoxifen-induced deletion of *Trp53* (Cre-loxP system). Analyses of experimental cohorts were performed at the age of 6, 12, 36, and 50 weeks to study inflammation and subsequent induction of liver fibrosis and tumor formation. (**C**) Bar graph represents the amount of *Trp53* deletion in whole liver DNA from 36 weeks old *Trp53^−/−^* (*n* = 3) and *Ikk2^ca^ Trp53^−/−^* mice (*n* = 4). As control whole liver DNA from *Trp53^+/+^* (*n* = 3) and *Ikk2^ca^* (*n* = 3) mice, and a positive control from a *Trp53* deleted primary cell line was used. Data represent the mean ±SD. (**D**) Western blots showing IKK2 expression of whole liver lysates from *Ikk2^ca^* and *Ikk2^ca^ Trp53^−/−^* mice at the age of 6, 12, 36, and 50 weeks. Whole liver lysates from 6 and 50 weeks old *Trp53^+/+^* mice were used as control. (**E**) Electrophoretic mobility shift assay (EMSA) depicting NF-κB activation and control (Oct1) of nuclear extracts from the liver of *Trp53^+/+^*, *Trp53^−/−^*, *Ikk2^ca^* and *Ikk2^ca^ Trp53^−/−^* mice at the age of 6, 12, 36, and 50 weeks. As control nuclear extracts from the liver of TNFα injected versus NaCl injected *Trp53^+/+^* mice were used. (**F**) Representative photographs of p65 immunohistochemistry (IHC) of liver tissue from *Trp53^+/+^*, *Trp53^−/−^*, *Ikk2^ca^* and *Ikk2^ca^ Trp53^−/−^* mice at the age of 6, 12, 36, and 50 weeks (scale bar: 50 µm). Arrows point at p65 positive nuclei.

To evaluate the role of NF-κB activation and p53 loss, we generated the following cohorts: (1) NF-κB activation in combination with p53 loss (*Ikk2^ca^*, *Trp53^−/−^*), (2) single NF-κB activation (*Ikk2^ca^*) [23], (3) single p53 loss (*Trp53^−/−^*) and (4) Cre positive mice without NF-κB activation and wildtype p53 allele (*Trp53^+/+^*) (Figure 1A). The mice were analyzed for inflammation, fibrosis and tumor formation at different time points (Figure 1B). With the liver-specific inducible CreERT2 system [24], we observed a *Trp53* gene deletion rate between 25% to 30% in *Trp53^−/−^* and *Ikk2^ca^ Trp53^−/−^* mice (Figure 1C). The western blot analysis of IKK2 revealed comparable expression levels in both *Ikk2^ca^* expressing cohorts independent of their p53 status (Figure 1D and Figures S1A,B and S7–S11). The highest IKK2^ca^ expression was observed at six weeks of age and declined slightly with increasing age (Figure 1D and Figure S7). IKK2^ca^ expression resulted in activation of the NF-κB signaling pathway, as evidenced by the nuclear NF-κB DNA-binding activity in electrophoretic mobility shift assay (EMSA), p65 nuclear staining and western blot analysis (Figure 1E,F and Figures S1A,B and S7–S11). Particularly, in week 6, we observed the highest NF-κB activation in *Ikk2^ca^* expressing mice with a drop-in activity at week 12 (Figure 1E,F). However, NF-κB activity returned at 36 weeks, indicating a biphasic mode of activation. Thus, expression of IKK2^ca^ led to increased NF-κB activation with a marked peak during the early period of IKK2^ca^ induction. The expression of a negative feedback regulator of NF-κB, *IL-10*, was decreased at week 6 and upregulated at week 12 in IKK2^ca^ expressing mice compared to controls. This could indicate a short-term cytoprotective response to an intense NF-κB activation, which declines upon IKK2^ca^ expression till the age of 36 weeks (Figure S1C).

### 2.2. IKK2^ca^ Expression Leads to Chronic Inflammation and Fibrosis Formation Independent of the p53 Status

**Figure 2:**
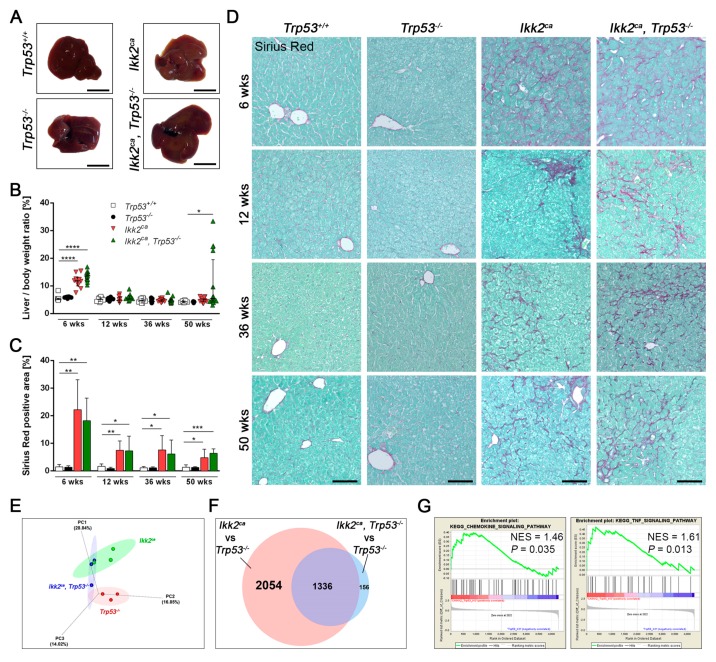
Induction of inflammation and liver fibrosis is independent of p53 status. (**A**) Macroscopic photographs of livers from 6 weeks old *Trp53^+/+^*, *Trp53^−/−^*, *Ikk2^ca^* and *Ikk2^ca^ Trp53^−/−^* mice (scale bar: 1 cm). (**B**) Liver/body weight ratio of *Trp53^+/+^*, *Trp53^−/−^*, *Ikk2^ca^* and *Ikk2^ca^ Trp53^−/−^* mice (*n* = 8–13). (**C**) Bar graph depict picro-sirius-red positive area in percentage (*n* = 5–8), data represent the mean ±SD; * *p* < 0.05, ** *p* < 0.01, *** *p* < 0.001, **** *p* < 0.0001. (**D**) Representative images of picro-sirius-red staining indicating liver fibrosis formation in *Trp53^+/+^*, *Trp53^−/−^*, *Ikk2^ca^* and *Ikk2^ca^ Trp53^−/−^* mice at all analyzed time points (scale bar: 100 µm). (**E**,**F**) Transcriptome analysis of pre-malignant liver tissue from 36 weeks old *Trp53^−/−^* (*n* = 3), *Ikk2^ca^* (*n* = 4) and *Ikk2^ca^ Trp53^−/−^* (*n* = 3) mice. (**E**) 3-D plot shows a principal components analysis of the entire dataset (the top 3 PCs explain ~60% of the cumulative variance). PC analysis depicts a partial overlap between *Ikk2^ca^* (green) and *Ikk2^ca^ Trp53^−/−^* (blue). (**F**) Venn diagram of the differential expression analysis showing overlap of 1336 differentially regulated genes between *Ikk2^ca^* and *Ikk2^ca^ Trp53^−/−^* as compared to the *Trp53^−/−^* control. (**G**) Gene Set Enrichment Analysis (GSEA) of chemokine (left) and TNF (right) signaling pathways in *Ikk2^ca^ Trp53^−/−^* vs. *Trp53^−/−^* mice.

Liver-specific expression of the IKK2^ca^ resulted in an inflammatory response upon NF-κB activation. Correlating with NF-κB peak activity, both *Ikk2^ca^* expressing cohorts exhibited a significantly higher liver weight and liver to body weight ratio at week 6 (Figure 2A,B and Figure S2A). The increased liver weight correlated with elevated infiltration of immune cells and increased cell proliferation again peaking at six weeks of age (Figure S2B–D). Among the immune cells analyzed, T-cells and neutrophils, were significantly enriched in the liver parenchyma during the whole observation period (Figure S2B). A significant upregulation of macrophages was detectable in liver parenchyma of *Ikk2^ca^* expressing mice at 6 and 12 weeks of age, independent of the p53 status (Figure S2B). In comparison to other immune cells, the number of B-lymphocytes was not altered in liver parenchyma. The extent of hepatic inflammation, as well as liver damage indicated by elevated ALT and AST levels, did not differ between six weeks old *Ikk2^ca^* and *Ikk2^ca^ Trp53^−/−^* mice, respectively (Figure S2E,F).

Liver inflammation resulted in marked fibrosis. Indeed, we observed liver fibrosis already in 6 weeks old *Ikk2^ca^* expressing mice. There was no change in fibrosis formation upon liver-specific deletion of *Trp53* (Figure 2C,D and Figure S2G). Thus, IKK2^ca^ expression results in inflammation, transaminitis and liver fibrosis, and is, therefore, an appropriate model to study the consequences of inflammatory signals in liver carcinogenesis.

To get further insights into the carcinogenic process, we did transcriptome analysis by using a whole genome gene expression microarray. We chose precancerous liver samples from 9-month old *Trp53^−/−^*, *Ikk2^ca^* and *Ikk2^ca^ Trp53^−/−^* mice for our analysis. We performed a principal component analysis (PCA), indicating an overlap between the *Ikk2^ca^* and *Ikk2^ca^ Trp53^−/−^* cohorts (Figure 2E). An analysis of differentially expressed genes (Figure 2F and Tables S1 and S2) revealed an overlap of 1336 genes between *Ikk2^ca^* and *Ikk2^ca^ Trp53^−/−^*, as compared to the *Trp53^−/−^* control. In addition, we observed an enrichment of inflammation-related pathways (Figure 2G).

### 2.3. Trp53 Deletion Leads to Increased Tumor Burden and Shift in Tumor Differentiation

**Figure 3:**
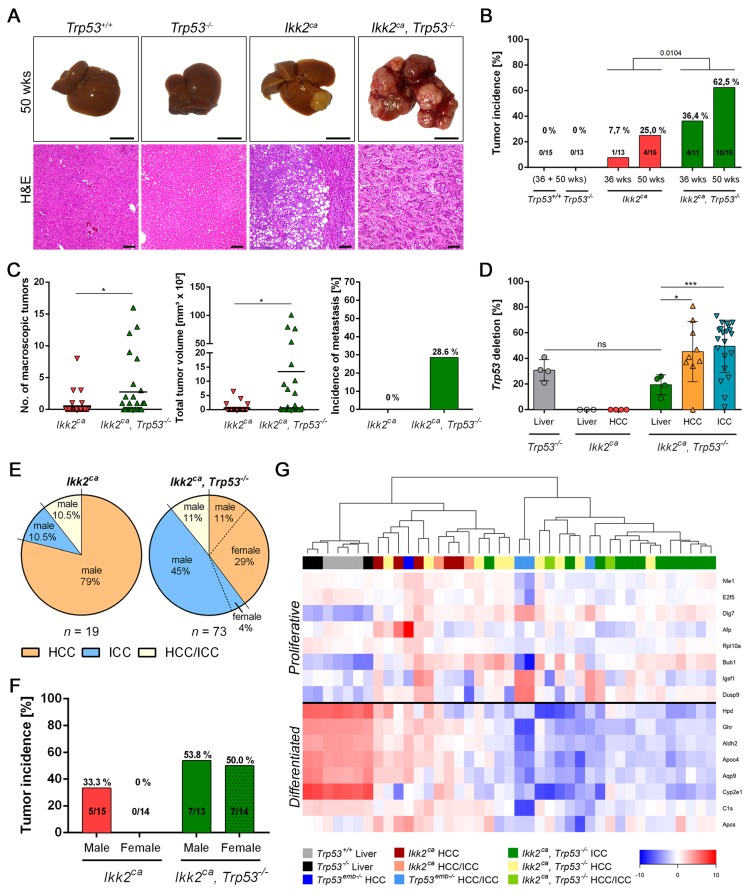
*Trp53* deletion results in increased tumor burden and modulates tumor differentiation in *Ikk2^ca^* expressing mice. (**A**) Representative macroscopic photographs (scale bar: 1 cm) and H&E sections (scale bar: 100 µm) of liver carcinoma in *Ikk2^ca^* and *Ikk2^ca^ Trp53^−/−^* mice. (**B**) Bar graphs represent liver tumor incidence. (**C**) Bar graph depicts tumor number, total tumor volume per mouse (*n* = 29 and 27) and incidence of metastasis (*n* = 5 and 14). The black line in scatter dot plots represent the mean. (**D**) Combined scatter dot plot bar graph represents the *Trp53* deletion rate in liver and liver carcinoma of the indicated genotype at the age of 50 weeks (*n* = 4–21). Bar graph of *Trp53* deletion represent the mean ±SD; * *p* < 0.05, ** *p* < 0.01, **** *p* < 0.0001. (**E**) Pie charts depict tumor differentiation of the indicated gender of *Ikk2^ca^* (*n* = 19) *and Ikk2^ca^ Trp53^−/−^* (*n* = 73) mice. (**F**) Gender-specific tumor incidence of *Ikk2^ca^ and Ikk2^ca^ Trp53^−/−^* mice. (**G**) Relative mRNA expression of a 16-gene signature of the indicated tissue and genotype.

Macroscopic tumor formation was observed in both *Ikk2^ca^* and *Ikk2^ca^ Trp53^−/−^* mice at the age of 36 weeks. However, *Trp53^−/−^* or *Trp53^+/+^* mice did not develop liver tumors within the analyzed time period (Figure 3A,B). Tumor incidence was increased in the *Ikk2^ca^ Trp53^−/−^* cohort and correlated significantly with the age of the mice (Figure 3B). Moreover, *Ikk2^ca^ Trp53^−/−^* mice developed more macroscopically visible liver tumors with a higher tumor volume (Figure 3C). In addition, we could detect metastasis in 29% of *Ikk2^ca^ Trp53^−/−^* tumor-bearing mice, mainly in the lung, pancreas and the diaphragm (Figure 3C and Figure S3A). We revealed an increased *Trp53* deletion rate in liver tumors compared to liver tissue (Figure 3D). This suggests an immediate contribution of *Trp53* deletion to tumor development. A histological evaluation and characterization, by CK7 and CK8/18 immunofluorescence, revealed different tumor entities (Figure 3E and Figure S3B,C). Both *Ikk2^ca^* expressing cohorts developed hepatocellular carcinoma (HCC), intrahepatic cholangiocarcinoma (ICC) and mixed differentiated carcinoma (HCC/ICC) at different ratios. Interestingly, we observed a marked shift in the tumor spectrum towards ICC formation in *Ikk2^ca^ Trp53^-/-^* mice (Figure 3E). In addition, we observed gender-specific differences in *Ikk2^ca^* mice. Among the analyzed animals, 5/15 (33.3%) male and 0/14 (0%) female *Ikk2^ca^* mice developed liver carcinoma. In contrast, we did not observe gender differences in *Ikk2^ca^ Trp53^−/−^* mice, where 7/13 (53.4%) male and 7/14 (50%) female *Ikk2^ca^ Trp53^−/−^* mice developed liver carcinoma (Figure 3F).

To further characterize the different tumor entities, a 16-gene profiling was performed to assess the rate of proliferation and the degree of differentiation [25]. RNA expression of *Ikk2^ca^*-induced tumors was compared to liver tissues and to liver tumors from an embryonically deleted *Trp53^emb−/−^* cohort [22]. *Ikk2^ca^ Trp53^−/−^* mice showed a high tumor heterogeneity with a high fraction of poorly differentiated liver carcinomas (Figure 3G).

### 2.4. Liver Progenitor Marker Persist in Liver Tumors of IKK2^ca^ Expressing Mice

**Figure 4:**
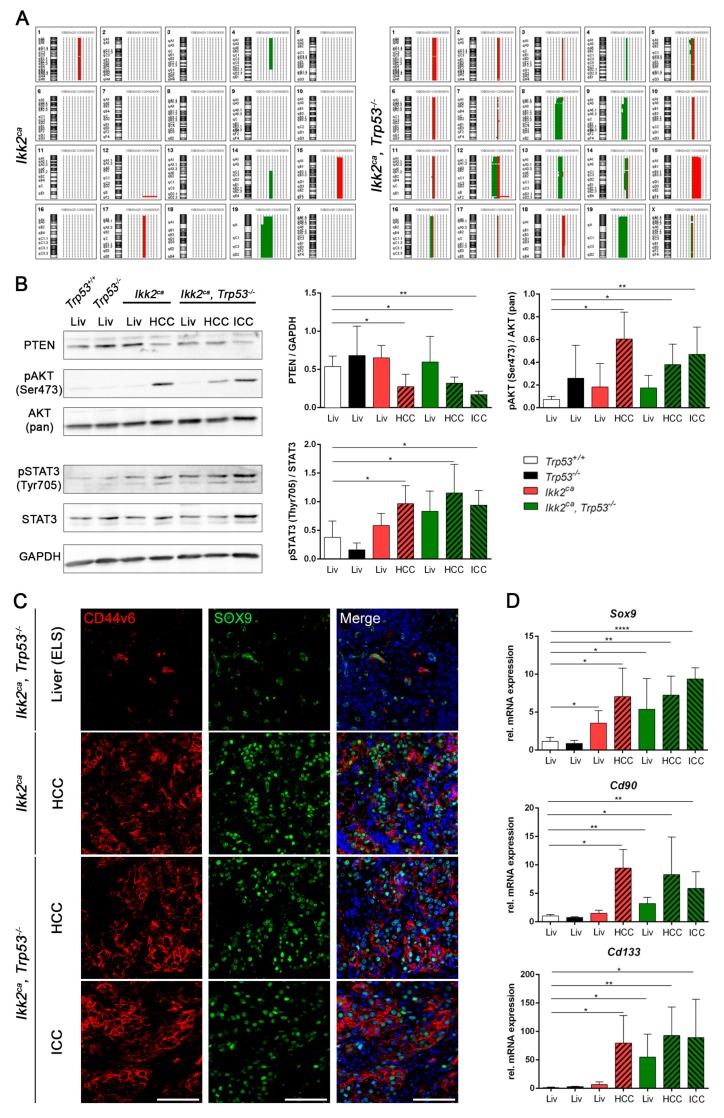
*Ikk2^ca^* dependent activation of pAKT and pSTAT upon PTEN downregulation promotes the formation of progenitor-like cells. (**A**) Ideogram summarizes genomic imbalances (gains, red; losses, green) in tumors derived from *Ikk2^ca^* (*n* = 6) and *Ikk2^ca^ Trp53^−/−^* (*n* = 14) mice. (**B**) Representative Western blots of liver/tumor from 50 weeks old mice of the indicated genotype and bar graphs representing quantification of relative protein level of PTEN/GAPDH (*n* = 4), pSTAT3/STAT3 (*n* = 4) and pAKT/AKT (*n* = 4). (**C**) Representative images of CD44v6 and SOX9-IF of liver (ELS—ectopic lymphoid structures) and liver carcinoma of *Ikk2^ca^*, *Ikk2^ca^ Trp53^−/−^* mice (scale bar: 100 µm). (**D**) Bar graphs represent relative mRNA expression of the stemness markers *Sox9*, *Cd90* and *Cd133* (*n* = 3–5). Data represent the mean ± SD; * *p* < 0.05, ** *p* < 0.01, **** *p* < 0.0001.

In respect to *Trp53* deletion, we performed aCGH to determine genomic imbalances in liver carcinomas. Expectedly, chromosomal instability was increased upon *Trp53* deletion (Figure 4A). A frequent loss of chromosome 8, 9 and 12 appeared upon *Trp53* deletion. Whereas gain of chromosome 15 and loss of chromosome 19 were present in both *Ikk2^ca^* expressing cohorts. A well-known tumor suppressor gene located at chromosome 19 is PTEN. We observed reduced PTEN protein levels, most likely as a result of the heterozygous loss of chromosome 19 (Figure 4A,B). We observed that the PTEN protein expression in *Ikk2^ca^ Trp53^−/−^* ICCs is negatively correlated with *Trp53* deletion rate (Figure S4A–C).

In addition, we have analyzed key components of the PI3K/AKT and STAT3 signaling pathways, which can be affected by dysregulated PTEN expression. The western blot analysis revealed increased amounts of pAKT and pSTAT3 in liver tumors of *Ikk2^ca^* expressing mice (Figure 4B and Figure S12).

**Figure 5:**
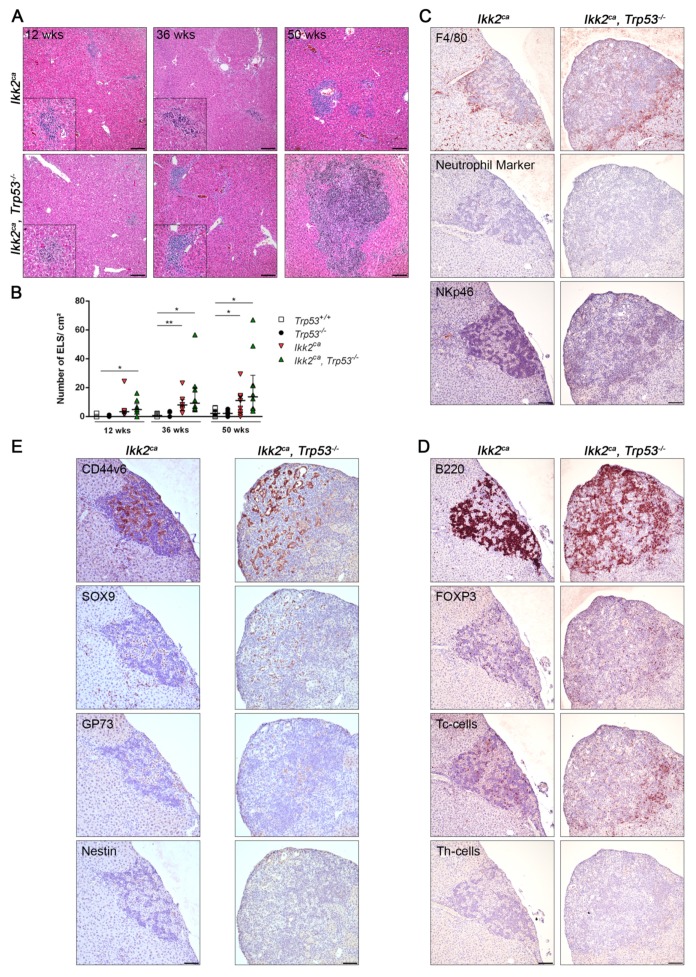
Formation of ectopic lymphoid structures (ELS) serves as a microniche for liver progenitor cells. (**A**) Representative photographs of H&E stained liver sections from *Ikk2^ca^* and *Ikk2^ca^ Trp53^−/−^* mice (scale bar: 100 µm). (**B**) Graph depicts the number of ELS per square centimeter in liver of *Trp53^+/+^*, *Trp53^−/−^*, *Ikk2^ca^* and *Ikk2^ca^ Trp53^−/−^* mice (*n* = 5–10). Data represent the median with interquartile range; * *p* < 0.05, ** *p* < 0.01. (**C**,**D**) Representative photographs of IHC for the innate immune system (**C**) macrophages (F4/80), neutrophils (Neutrophil Marker) and NK cells (NKp46) and the adaptive immune system (**D**) B-cells (B220), T regulatory cells (FOXP3), cytotoxic T-cells (Tc-cells) and T helper-cells (Th-cells) in ELS of *Ikk2^ca^* and *Ikk2^ca^ Trp53^−/−^* mice (scale bar: 100 µm). (**E**) Accumulation of liver progenitor cells in ELS. Representative photographs of IHC for CD44v6, SOX9, GP73 and Nestin in ELS of *Ikk2^ca^* and *Ikk2^ca^ Trp53^−/−^* mice (scale bar: 100 µm). The IHC are performed on consecutive sections for immune cells (**C**,**D**) and progenitor cell markers (**E**).

Activation of STAT3 has been linked to upregulation of progenitor cell markers in HCC [26]. We observed a frequent accumulation of cells expressing the progenitor markers CD44v6 and SOX9 in *Ikk2^ca^*-induced tumors (Figure 4C). In addition, there were increased RNA expression levels of the progenitor cell markers *Sox9*, *Cd90* and *Cd133* in *Ikk2^ca^*-induced tumors and in liver tissue of *Ikk2^ca^* and *Ikk2^ca^ Trp53^−/−^* mice (Figure 4D). In premalignant liver tissue, accumulation of progenitor cells has been reported within so-called ectopic lymphoid structures (ELS) [27]. These aggregates are characterized by a cluster formation of infiltrating immune cells function as a microniche for tumor initiating cells [27]. Here we demonstrate that expression of IKK2^ca^ resulted in the formation of ELS in the liver (Figure 5A). The number of ELS increased with the age of the mice, and there was comparatively a higher number of ELS in the liver of *Ikk2^ca^ Trp53^−/−^* mice (Figure 5A,B). Detailed analysis revealed that ELS consisted mainly of macrophages, T- and B-cells and neutrophils (Figure 5C,D and Figure S5A,B). Furthermore, we detected the accumulation of progenitor-like cells within the ELS that were positive for CD44v6, GP73, SOX9 and Nestin (Figure 5E and Figure S6A–C). These results are in line with the observation that a higher frequency of markers related to stemness in liver tissue appears first within the ELS. Those progenitor-like cells might be the tumor initiating cells [27].

### 2.5. Differentially Regulated Genes in Liver Carcinogenesis of Ikk2^ca^ Expressing Mice

**Figure 6:**
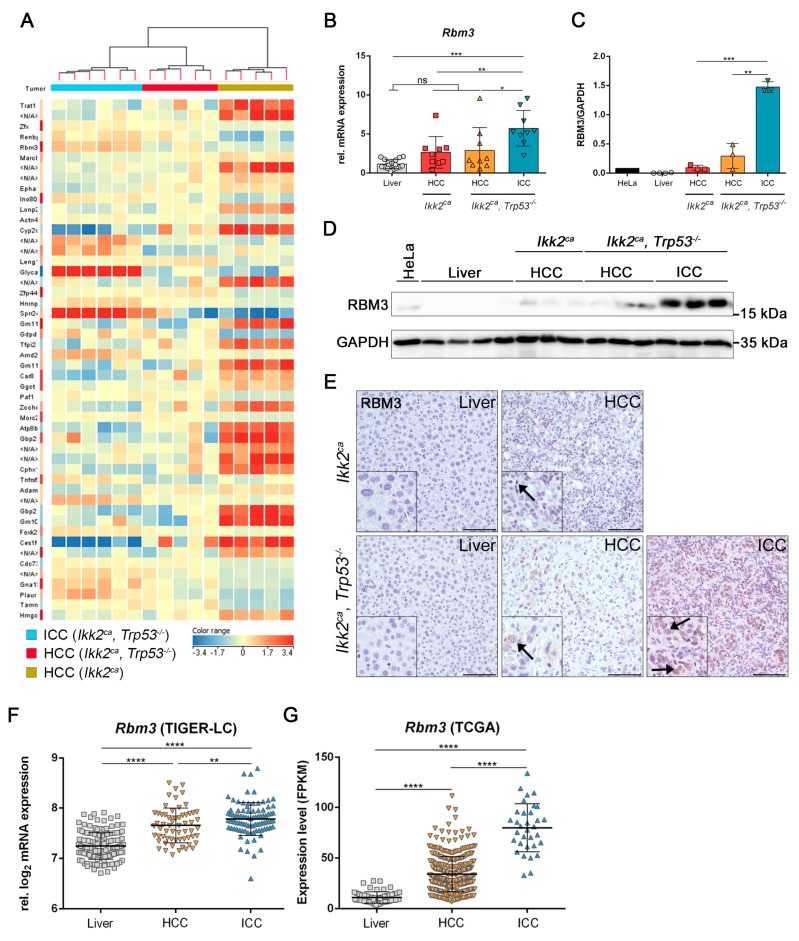
Transcriptome analysis of *Ikk2^ca^* derived tumors. (**A**) Heatmap of expression profiles of the indicated group. (**B**) Combined scatter dot plot bar graph represents relative mRNA expression of *Rbm3* in liver tissue from *Trp53^+/+^*, *Trp53^−/−^*, *Ikk2^ca^ and Ikk2^ca^ Trp53^−/−^* mice (*n* = 3–5/genotype), HCC (*n* = 9) form *Ikk2^ca^* mice and HCC (*n* = 9), ICC (*n* = 9) from *Ikk2^ca^ Trp53^−/−^* mice. (**C**,**D**) Graphs represent quantification (**C**) and western blot analysis (**D**) of RBM3 protein level in liver tissue from *Ikk2^ca^* (*n* = 2) *and Ikk2^ca^ Trp53^−/−^* (*n* = 2) mice, HCC (*n* = 3) form *Ikk2^ca^* mice and HCC (*n* = 3), ICC (*n* = 3) from *Ikk2^ca^ Trp53^−/−^* mice. Protein lysate from HeLa cells was used as a positive control. (**E**) Representative images of RBM3 IHC in adjacent liver tissue and liver tumors from the *Ikk2^ca^ and Ikk2^ca^ Trp53^−/−^* mice. Arrows point to RBM3 positive cells (scale bar: 100 µm). (**F**) Graphs represent relative gene expression values of *Rbm3* in non-tumorous liver tissue (*n* = 151), HCC (*n* = 62) and ICC (*n* = 91) from the TIGER-LC. (**G**) Graphs represent relative gene expression values of *Rbm3* in non-tumorous liver tissue (*n* = 58), HCC (*n* = 371) and ICC (*n* = 33) from the TCGA database (TCGA-LIHC and TCGA-CHOL). In (**F**) and (**G**) Mann-Whitney test was performed. All data represent the mean ± SD; * *p* < 0.05, ** *p* < 0.01, *** *p* < 0.001, **** *p* < 0.0001.

In order to identify tumor type-specific genes that could be important for increased tumor burden and the modulation of tumor differentiation, we performed a whole-genome gene expression microarray analysis of liver tumors from *Ikk2^ca^* mice and *Ikk2^ca^ Trp53^−/−^* mice, respectively. We identified 37 differentially regulated genes by a one-way ANOVA test (Figure 6A and Table S3). This analysis also demonstrated the high heterogeneity of liver tumors within the *Ikk2^ca^ Trp53^−/−^* group with a clear distinction of HCCs and ICCs within this subgroup (Figure 6A). One of the most upregulated genes in ICCs in the *Ikk2^ca^ Trp53^−/−^* group was RNA-binding Motif Protein 3 (*Rbm3)*, a key regulator of transition in cell morphology (Figure 6A). Higher *Rbm3* expression was confirmed by qPCR, western blot analysis and immunohistochemistry (Figure 6B–E and Figure S13). RBM3 has been reported to be involved in carcinogenesis [28,29]. Data from the TIGER-LC and TCGA showed a similar expression pattern for *Rbm3* in adjacent human liver, HCC and ICC samples, respectively, when compared to our murine data (Figure 6F,G). Evidently, higher levels of *Rbm3* are associated with ICC in human and in mouse (Figure 6B–G and Figure S13).

## 3. Discussion

Our study describes and characterize a transgenic model demonstrating the consequences of NF-κB activation and the subsequent loss of p53. Here we used inducible Tet-Off and Cre mouse models, which determines the precise start of IKK2^ca^ expression in hepatocytes and *Trp53* deletion in liver parenchymal cells. In this way, we could modulate in a time adjusted manner two major hallmarks of liver carcinogenesis. This inducible system resembles human liver carcinogenesis more closely with p53 mutations occurring only in some initiating cells, rather than in the whole liver.

In HCC, the NF-κB pathway acts as a double-edged sword. It has been described as a tumor promoter [17,19,27], but also as a tumor suppressor [18,19]. In addition, the activation/inhibition of NF-κB in immune cells interferes with tumor formation [18]. The *Mdr2* knockout mouse, a model for spontaneous cholestatic hepatitis, revealed activation of NF-κB followed by HCC formation [17]. Here, inhibition of the NF-κB activation in hepatocytes resulted in an increased tumor formation [17]. Conversely, depletion of *Ikk2* in hepatocytes in a diethylnitrosamine (DEN)-induced HCC mouse model resulted in increased tumor formation, which is disturbed by *Ikk2* deletion in hematopoietic-derived Kupffer cells [18]. These results depict the importance of the crosstalk between hepatocytes and hematopoietic-derived cells in the DEN model. However, liver-specific deletion of *Ikk2* did not lead to spontaneous liver disease [19]. Activation of the NF-κB pathway via expression of IKK2^ca^ in hepatocytes resulted in macrophage-mediated inflammation and fibrosis in *LAPtTA Ikk2^ca^* mice, or formation of ELS with a very late onset of liver tumors at the age of 20 months in Alb-Cre x *R26Stop*^FL^*Ikk2ca* mice [23,27].

Gender-specific differences are described in inflammation-driven models of liver cancer. The chemical carcinogen DEN leads to increased IL-6 production and induces HCC in 100% of male mice, but only in 10–30% of female mice [18,30]. In contrast, the knockout of IL-6 results in impaired HCC development in male mice, whereas, in females, no differences were detected between IL-6 wildtype and IL-6 knockout mice [31]. The administration of estrogens inhibits DEN-induced carcinogenesis and IL-6 production [30,31]. In alignment with previous findings, almost exclusively male mice from the *Ikk2^ca^* cohort developed liver carcinomas (male: 5/15; female: 0/14). Remarkably, deletion of *Trp53* in *Ikk2^ca^* expressing mice abolished this effect, leading to related tumor development in both genders (male: 7/13; female: 7/14).

In accordance with previous findings, we could detect the formation of ELS upon activation of IKK/NF-κB signaling [27]. In patients, these focal immune cell clusters have been described to have anti-tumorigenic properties in certain solid tumors like lung cancer [32]. In the liver, the function of ELS is rather controversial. ELS have been described as conferring effective anti-tumor immunity [33], but were also associated with poor prognosis in patients with HCC [27]. In our model, we observed a strong tendency of IKK2^ca^ expressing mice to form ELS harboring progenitor-like cells with evidence to tumor egress, strongly supporting a pro-tumorigenic role of these structures. It remains to be elucidated, whether ELS promote malignant cell transformation or have anti-tumorigenic effects on already established tumor initiating cells. The occurrence of the progenitor-like cells was p53-independent. This may be due to the fact that NF-κB and p53 can inhibit each other’s ability to stimulate gene expression, explained by interaction with the same transcriptional coactivator proteins [34].

We observed a frequent loss of chromosome 19 in both *Ikk2^ca^* expressing cohorts. This suggests that the loss of chromosome 19 could be linked to inflammation-driven liver carcinogenesis. PTEN, which is located on chromosome 19, was downregulated in *Ikk2^ca^* expressing mice at the protein level. Two recent publications of a KRAS activation mouse model, also a common aberration in ICC, showed that *Pten^−/−^* mice exclusively developed ICCs, whereas *Pten^+/−^* mice formed ICC and HCC [35] or mixed differentiated tumors [36]. Similarly, in our mouse model there is a strong negative correlation between PTEN expression and *Trp53* deletion rate in ICCs, which might represent an important event for the shift towards ICC formation in *Ikk2^ca^ Trp53^−/−^* mice. Furthermore, it has been reported, that PTEN can regulate AKT and STAT3 signaling in neural cancer stem cells. While overexpression of PTEN led to reduced phosphorylation of AKT and STAT3, the inhibition of PTEN shows the opposite effect [37]. It becomes even more interesting considering that STAT3 signaling has been described to be responsible for the growth of stem-like cancer cells in different tumors and induce the expression of the stem cell marker CD133 [26,38], similar to our observation in *Ikk2^ca^* expressing mice.

We could detect *Rbm3* upregulation in *Ikk2^ca^* derived tumors, which was even higher upregulated in ICC tumors. *Rbm3* was also upregulated in two independent cohorts of human liver carcinomas, especially in ICCs. *RBM3* has been reported to promote the development of colitis-associated cancer or enhance migration ability in neuroblastoma cell lines [28,29]. In addition, high *RBM3* levels were significantly associated with reduced overall survival in pancreatic cancer [39]. It still remains to be evaluated whether the RBM3 has a regulatory function in differentiation towards ICC formation or is a bystander effect.

## 4. Material and Methods

### 4.1. Mouse Model

To generate mice with a liver-specific inflammatory response, we bred mice carrying the LAPtTA allele to mice with a constitutively active human *Ikk2* allele under the control of a tetracycline response element promoter [23]. All *Ikk2^ca^* bearing mice received 0.1 g/L doxycycline in drinking water until birth to avoid embryonic activation of the NF-κB pathway. Upon birth, doxycycline treatment was immediately stopped to induce a liver-specific IKK2^ca^ expression. Conditional B6.129P2-*Trp53^tm1Brn^*/J mice (JAX stock #008462) [40] were crossed with *Alfp-CreERT2* transgenic mice [24] to generate inducible liver-specific *Trp53* knockout mice. At the age of four weeks, the mice were injected intraperitoneally with 125 mg/kg tamoxifen (T5648-5G, Sigma-Aldrich, Saint Louis, MO, USA) dissolved in ethanol and sunflower oil for five consecutive days to induce liver-specific deletion of *Trp53*. Mice were analyzed at the age of 6, 12, 36, and 50 weeks.

The following cohorts were generated and utilized in this study: (1) LAPtTA Ikk2^ca^ AlfpCre-ERT2^+^ Trp53^fl/fl^ (Ikk2^ca^ Trp53^−/−^), (2) LAPtTA Ikk2^ca^ (Ikk2^ca^), (3) AlfpCre-ERT2^+^ Trp53^fl/fl^ (Trp53^−/−^) and (4) AlfpCre-ERT2^+^ Trp53^+/+^ (Trp53^+/+^).

The mice were maintained in a specific pathogen-free environment. All animal experiments were approved by the state government of Baden-Württemberg (protocol number 35/9185.81-3/1175).

### 4.2. Liver Histology

Liver tissue samples were fixed in 4% paraformaldehyde (PFA) overnight at 4 °C, dehydrated in ethanol and xylene, and embedded in paraffin. Standard protocols were used for hematoxylin and eosin (H & E) staining on 3-µm-thick paraffin sections and for Picro Sirius Red staining on 5-µm-thick paraffin sections, respectively. For measuring the Sirius Red positive area ImageJ software (v.1.47, Rasband, W.S., ImageJ, U. S. National Institutes of Health, Bethesda, Maryland, MD, USA, https://imagej.nih.gov/ij/, 1997-2018.) with color deconvolution plugin was used.

### 4.3. Immunohistochemistry and Immunofluorescence

For immunohistochemistry and immunofluorescence, 5-µm-thick paraffin-embedded liver sections were deparaffinized and rehydrated. Antigen retrieval for F4/80 and Neutrophil Marker was achieved by incubation of liver sections with 20 µg/mL recombinant Proteinase K (Merck Millipore, Burlington, MA, USA) for 10 min at room temperature (RT). Heat mediated antigen retrieval for Ki67, p65, B220, FOXP3, CD8, CD4, NKp46, CD44v6, SOX9, GP73, Nestin and RBM3 was performed using antigen-unmasking solution (Vector Laboratories, Burlingame, CA, USA) and for CK7, CK8/18 using EDTA buffer (Thermo Scientific, Waltham, MA, USA) in a steamer for 35 min. Liver sections were incubated with primary antibodies at 4 °C overnight or at RT for 60 min. The primary antibodies used in experiments are listed in Additional file 4: Table S4. Corresponding biotinylated or fluorophore-conjugated secondary antibodies were applied at RT for 60 or 90 min. Samples with biotinylated secondary antibody were detected using Nova red (Vector Laboratories) and counterstained with 20% hematoxylin. Mounting medium with DAPI (Vector Laboratories) was used to stain nuclei for samples with fluorophore-conjugated secondary antibody.

### 4.4. Genomic DNA Isolation and Analysis of Trp53 Deletion Rate

Genomic DNA was isolated from liver and tumor tissue according to standard phenol-chloroform extraction protocol for *Trp53* deletion PCR or using the DNeasy Blood and Tissue Kit (Qiagen, Valencia, CA, USA) for aCGH. Quantitative real-time PCR was performed to amplify genomic DNA with specific primers, detecting only deleted or floxed *Trp53,* as previously described [22].

### 4.5. Western Blotting Analysis

For whole-cell lysate, frozen liver tissue was homogenized in RIPA buffer (50 mM TrisHCl pH 8, 150 mM NaCl, 1% NP-40, 0.5% DOC, 0.1% SDS, 1 mM NaVO_3_, 1 mM DTT, 1 mM PMSF) containing protease inhibitors (Roche Diagnostics, Mannheim, Germany). The lysate was centrifuged at 15,000 g for 20 min at 4 °C, and the supernatant was collected. Freshly isolated proteins were snap-frozen and stored at −80 °C until further use. The protein concentration was determined using a Bradford assay (Bio-Rad, Hercules, CA, USA). Protein extracts (20 µg) were separated by SDS-PAGE and subsequently semi-dry blotted on a PVDF membrane. Membranes were blocked in 5% BSA/TBS-T or 5% milk/TBS-T and incubated with primary antibodies overnight at 4 °C. Primary antibodies are listed in Additional file 4: Table S4. Bands were detected after labeling the blot with an appropriate secondary HRP-conjugated antibody and incubation in the chemiluminescent HRP substrate (Merck Millipore). For protein stripping membranes were incubated for 30 min at 50 °C in stripping buffer (6.21 M Tris-HCl pH 6.8, 20 mL 10% SDS, 700 µL 2-mercaptoethanol and 73.05 mL deionized H_2_O), washed 3 × 10 min in TBS-T and blocked prior incubation with primary antibody.

### 4.6. RNA Isolation and cDNA Synthesis

Isolation of total RNA from snap-frozen liver or tumor tissue was performed with the RNeasy Mini Kit (Qiagen) according to the manufacturer’s protocol. Synthesis of complementary DNA for quantitative real-time PCR analysis was performed using the Reverse Transcription System (Promega, Madison, WI, USA) following the manufacturer’s protocol.

### 4.7. Quantitative Real-time PCR and 16-gene Set Analysis

For the amplification of cDNA an iTaq Universal SYBR Green Supermix (Bio-Rad) was used on an ABI 7500 Real-Time PCR System (Applied Biosystems, Foster City, CA, USA). The relative mRNA expression levels were calculated by the ΔΔCt method using RNA Polymerase II expression levels for normalization and age-matched *Trp53^+/+^* livers as calibrator. The primer sequences used are listed in Additional file 5: Table S5.

For the 16-gene set analysis [25] mRNA expression levels were normalized to RNA Polymerase II and set in relation to the mean expression of all samples. Heatmap was generated by an unsupervised hierarchical clustering using R software (v.3.2.3, https://www.R-project.org/) and gplots package. The primer sequences for the 16-gene set are listed in Additional file 5: Table S5.

### 4.8. Serum Parameters

Immediately after euthanization of mice, blood was collected from the *vena cava* and transferred into a Multivette 600 blood collection system (Sarstedt, Nümbrecht, Germany). After gently mixing, blood was centrifuged at 10,000 g for 5 min at 20 °C. Serum was collected in a fresh tube, snap-frozen and stored at −80 °C. Levels of enzymes indicating liver damage, alanine aminotransferase (ALT) and aspartate aminotransferase (AST), were measured in serum using a Reflotron system (Roche Diagnostics).

### 4.9. Electrophoretic Mobility Shift Assay (EMSA)

For the isolation of nuclear protein fraction, frozen liver tissue was homogenized in Dignam A buffer (10 mM HEPES pH 7.9, 1.5 mM MgCl_2_, 10 mM KCL, 50 mM PMSF, 1 mM DTT and protease inhibitor) and centrifuged at 5000 rpm for 10 min at 4 °C. The pellet was washed in Dignam A with 0.1% Triton X-100, centrifuged and re-suspended in Dignam C buffer (20 mM HEPES pH 7.9, 25% glycerol, 0.42 M NaCl, 1.5 mM MgCl_2_ and 0.2 mM EDTA, 1 mM DTT, 1 mM PMSF, 1 mM Na_3_VO_4_ and protease inhibitor). The protein solution was incubated for 1 h at 4 °C and finally centrifuged at 13,000 rpm for 20 min at 4 °C. The supernatant containing nuclear proteins was collected and used in a Bradford assay to determine protein concentration. Nuclear proteins (2–6 µg) were used in EMSA experiments, as previously described [41].

### 4.10. Array-based Comparative Genomic Hybridization (aCGH)

Agilent oligonucleotide array-based CGH for genomic DNA analysis (SurePrint G3 Mouse CGH Microarray Kit 4 × 180K, Agilent Technologies, Santa Clara, CA, USA) was performed on frozen tumor tissue. One microgram of the isolated genomic DNA was digested and labeled by random primer with Cyanine 5-dUTP using the SureTag DNA Labeling Kit (Agilent Technologies) according to the manufacturer’s protocol. As reference, a spleen genomic DNA from C57BL/6 mice was used. Microarray slides were scanned using a G2565CA microarray scanner (Agilent Technologies), and raw data were extracted using the Feature Extraction software (v.10.7.3.1, Agilent Technologies) for further analyzes with the Genomic Workbench software (v.7.0.4.0, Agilent Technologies). The extracted files were analyzed with the statistical algorithm ADM-2.

### 4.11. Gene Expression Analysis

Total RNA was isolated from whole liver or tumor tissue, and the quality was analyzed by the bioanalyzer (Agilent Technologies). Samples with a RIN (RNA integrity number) value higher than 8.0 were used for further processing. Gene expression analysis was carried out using the Mouse GE 4x44Kv2 Microarray Kit (Design ID 026655; Agilent Technologies) for liver samples and SurePrint G3 Mouse Gene Expression 8 × 60K Microarray Kit for tumor samples (Design ID 028005; Agilent Technologies). Samples were labeled with the Low Input Quick Amp Labeling Kit (Agilent Technologies) according to the manufacturer’s guidelines. Slides were scanned using a G2565CA microarray scanner (Agilent Technologies). Raw data files were extracted using the Feature Extraction software (v.10.7.3.1, Agilent Technologies, Santa Clara, CA, USA). For gene expression data generated from liver tissue Principal Component Analysis (PCA) was performed using R statistical package [42]. Quantile normalized, and log transformed gene expression values were used for the analysis. The top 3 principal components (PCs) to generate the 3D plot. Differential expression analysis was performed using the R and Bioconductor package limma [43]. Venn diagrams were generated using custom scripts in R. Gene Set Enrichment Analysis (GSEA) was performed using the standalone GSEA java application [44]. Correlation between GSEA and correlation plots were generated using in-house custom R scripts. The extracted raw data files from tumor samples were further processed by the GeneSpring GX software (Agilent Technologies). The analysis was performed based on Agilent´s Biological Significance guided workflow. The raw expression files were normalized, including log2 transformation, normalization to 75% quantile and normalization to the median. Afterwards, the data were filtered by flags. Differentially expressed genes were determined by ANOVA statistics corrected for multiple testing with False Discovery Rate (FDR < 0.1), following a post hoc test Tukey’s HSD (honestly significant difference). Hierarchical clustering with Euclidean distance and Ward´s linkage was performed on differentially expressed genes.

### 4.12. Gene Expression Analysis of Human Data

The human data set from the Thailand Initiative in Genomics and Expression Research for Liver Cancer (TIGER-LC) consist out of HCC (*n* = 62), HCC-adjacent non-tumor tissue (*n* = 59), ICC (*n* = 91) and ICC-adjacent non-tumor tissue (*n* = 92). The data set is available on Gene Expression Omnibus (GSE76297) and provides normalized log2 gene expression data [45]. Furthermore, a second, independent data set was used from the The Cancer Genome Atlas (TCGA) which includes HCC (*n* = 371), HCC-adjacent non-tumor tissue (*n* = 50), ICC (*n* = 33) and ICC-adjacent non-tumor tissue (*n* = 8). The data sets used in this study are available on TCGA-LIHC and TCGA-CHOL and provide gene expression values (FPKM). *Rbm3* gene expression values were extracted (TIGER-LC, TCGA-LIHC and TCGA-CHOL) and assessed with GraphPad Prism (v 6.04; GraphPad Software, Inc., La Jolla, CA, USA) using Mann-Whitney test to detect significant differences between the single groups.

### 4.13. Statistical Analysis

Statistical significance was assessed with GraphPad Prism (v 6.04, GraphPad Software, Inc.) using two-tailed Student *t* test (with Welch´s correction), chi-squared test and Fisher exact test. For human gene expression data, a Mann-Whitney test was used. The data are expressed as the median with interquartile range or mean ± SD, as indicated in figure legends. *p* < 0.05 was considered statistically significant.

## 5. Conclusions

Together the current study provides experimental evidence and an experimental model demonstrating that the inflammatory environment is an important driver in hepatocarcinogenesis, leading to the formation of different tumor entities. In addition, we provide the first experimental results for the interaction of p53 loss and IKK/NF-κB driven inflammation. Moreover, loss of p53 results in increased tumor burden and metastasis, a shift in tumor differentiation towards ICC formation and increased chromosomal aberrations. Similar aberration patterns between HCC and ICC link the appearance of these two tumor entities to a common tumor initiating cell, which requires different regulatory mechanisms for tumor differentiation other than chromosomal aberrations.

## Supplementary Materials

The following are available online at https://www.mdpi.com/2072-6694/11/10/1410/s1. Figure S1: Analysis of the NF-kB pathway and IL10 in Ikk2ca expressing mice. (Related to Figure 1), Figure S2: Induction of inflammation and liver fibrosis is independent of p53 status. (Related to Figure 2), Figure S3: Metastasis and tumor differentiation, Figure S4: Correlation of PTEN expression with *Trp53* deletion status in *Ikk2^ca^ Table.* Mice, Figure S5: immunohistochemistry (IHC) of immune cells in ectopic lymphoid structures (ELS), Figure S6: IHC of liver progenitor cells in ectopic lymphoid structures (ELS), Figure S7: Whole length western blots (of western blots, shown in Figure 1D), and densitometry readings/intensity ratios, Figure S8: Whole length western blots (of western blots, shown in Figure S1A), and densitometry readings/intensity ratios, Figure S9: Whole length western blots (of western blots, shown in Figure S1A, continued), and densitometry readings/intensity ratios, Figure S10: Whole length western blots (of western blots, shown in Figure S1B), and densitometry readings/intensity ratios, Figure S11: Whole length western blots (of western blots, shown in Figure S1B, continued), and densitometry readings/intensity ratios, Figure S12: Whole length western blot (of western blots, shown in Figure 4B), and densitometry readings/intensity ratios, Figure S13: Whole length western blot (of western blots, shown in Figure 6D), and densitometry readings/intensity ratios, Table S1: Transcriptome analysis: *Ikk2^ca^* vs. *Trp53^−/−^*. Table is showing statistics of transcriptome analysis from the comparison of liver from *Ikk2^ca^* vs. liver from *Trp53^−/−^* mice, Table S2: Transcriptome analysis: *Ikk2^ca^ Trp53^−/−^* vs. *Trp53^−/−^*. Table is showing statistics of transcriptome analysis from the comparison of liver from *Ikk2^ca^ Trp53^−/−^* vs. liver from *Trp53^−/−^* mice, Table S3: Significance Analysis of gene expression data from liver carcinoma generated in *Ikk2^ca^* and *Ikk2^ca^ Trp53^−/−^* mice. Table is showing one-way ANOVA analysis of gene expression data from liver carcinoma of *Ikk2^ca^* and *Ikk2^ca^ Trp53^−/−^* mice, Table S4: Antibodies. Table presents a list of antibodies used for IHC/IF and western blot, Table S5: Primers. Table presents primer names and sequences used for qRT-PCR and for the 16-gene set with proliferative and differentiated gene signature.
