# Supplementary material for: An IKK/NF-κB Activation/p53 Deletion Sequence Drives Liver Carcinogenesis and Tumor Differentiation
Source: Cancers (Basel). 2019 Sep 21;11(10):1410. doi: 10.3390/cancers11101410 (PMC6827060; doi:10.3390/cancers11101410)
Supplement: Supplementary file 1 [file cancers-11-01410-s001.zip › cancers-584517-supplementary.docx]

Supplementary Materials: An IKK/NF-κB Activation/p53 Deletion Sequence Drives Liver Carcinogenesis and Tumor Differentiation

**Michael Svinarenko, Sarah-Fee Katz, Umesh Tharehalli, Medhanie A. Mulaw, Harald J. Maier, Yoshiaki Sunami, Sarah K. Fischer, Yuexin Chen, Sabine Heurich, Lena Erkert, Andrea Tannapfel, Thomas Wirth, Reinhold Schirmbeck, Thomas Seufferlein and André Lechel**


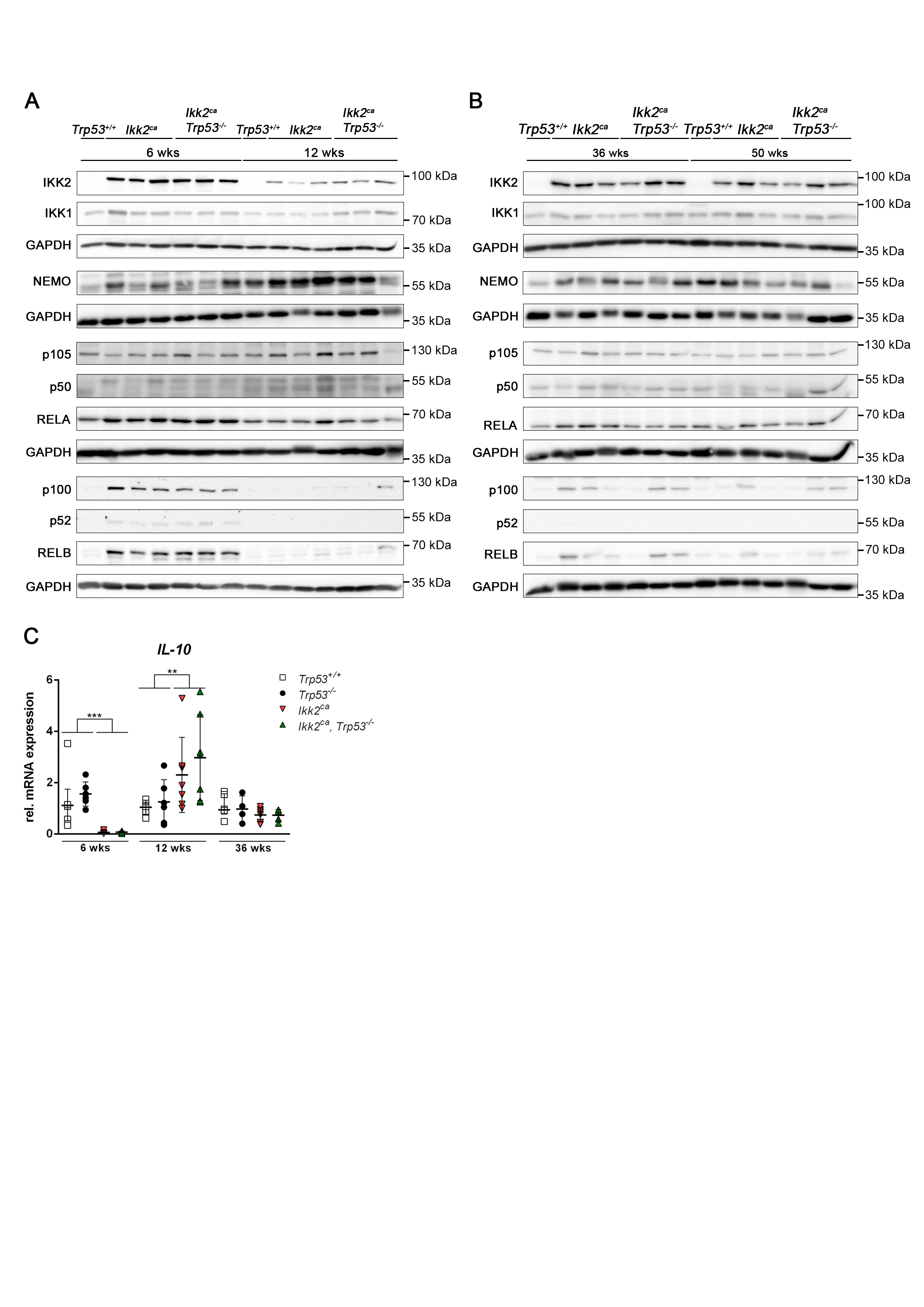


**Figure S1.** Analysis of the NF-kB pathway and IL10 in *Ikk2^ca^* expressing mice. (Related to Figure 1). (**A,B**) Western blots showing expression of IKK and NF-κB subunits of whole liver lysates from *Ikk2^ca^* (*n* = 3) and *Ikk2^ca^ Trp53^−/−^* (*n* = 3) mice at the age of (**A**) 6 and 12, (**B**) 36 and 50 weeks. Whole liver lysates from age matching *Trp53^+/+^* mice were used as control. (**C**) Scatter dot plot represents relative mRNA expression of *IL-10*, a negative feedback regulator of NF-κB, in liver of *Trp53^+/+^*, *Trp53^−/−^*, *Ikk2^ca^* and *Ikk2^ca^ Trp53^−/−^* mice (*n* = 4–7). *IL-10* expression is suppressed in 6 weeks old *Ikk2^ca^* expressing mice. Data represent the mean ± SD; ** *p* < 0.01, *** *p* < 0.001.


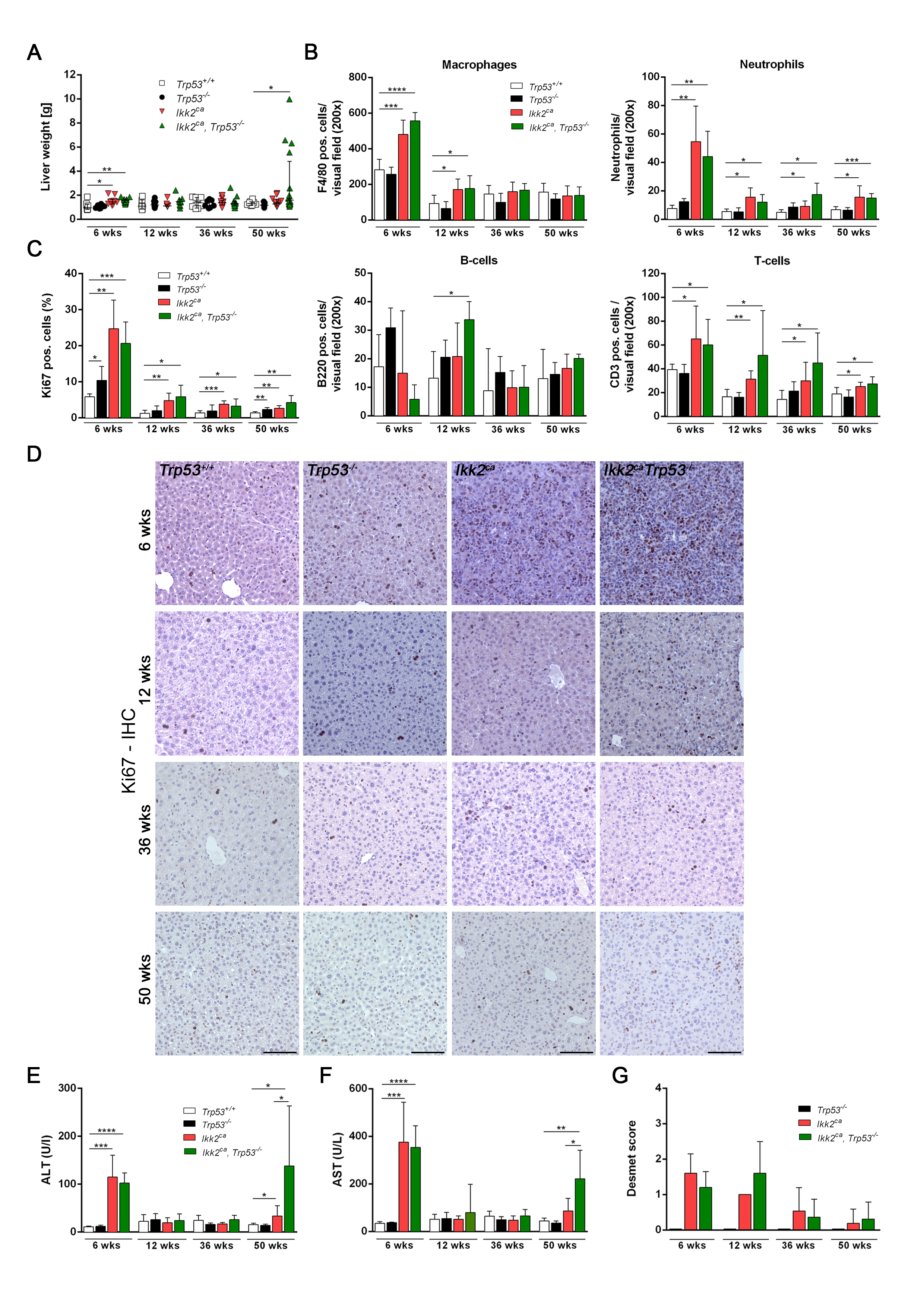


**Figure S2.** Induction of inflammation and liver fibrosis is independent of p53 status. (Related to Figure 2). (**A**) Scatter dot plot shows liver weight of *Trp53^+/+^*, *Trp53^−/−^*, *Ikk2^ca^* and *Ikk2^ca^ Trp53^−/−^* mice (*n* = 8–13) at the indicated age. (**B**) Bar graphs show absolute numbers of immune cells per visual field (200x): macrophages, neutrophils, B-cells and T-cells (*n* = 5–9). Immune cells within ELS were excluded from this analysis. (**C**) Bar graph depicts relative number of Ki67 positive cells (*n* = 5–7). Data represented as mean ±SD, * *p* < 0.05, ** *p* < 0.01, *** *p* < 0.001, **** *p* < 0.0001. (**D**) Representative images of Ki67-IHC indicating increased cell proliferation in *Ikk2^ca^* expressing mice (scale bar: 100µm). (**E,F**) Bar graphs represent ALT (alanine transaminase) and AST (aspartate transaminase), measured in mouse serum (*n* = 5–9) indicating liver damage in *Ikk2^ca^* expressing mice. (**G**) Bar graph depict Desmet Score (*n* = 3–16) in *Trp53^−/−^*, *Ikk2^ca^* and *Ikk2^ca^ Trp53^−/−^* mice at the indicated age.


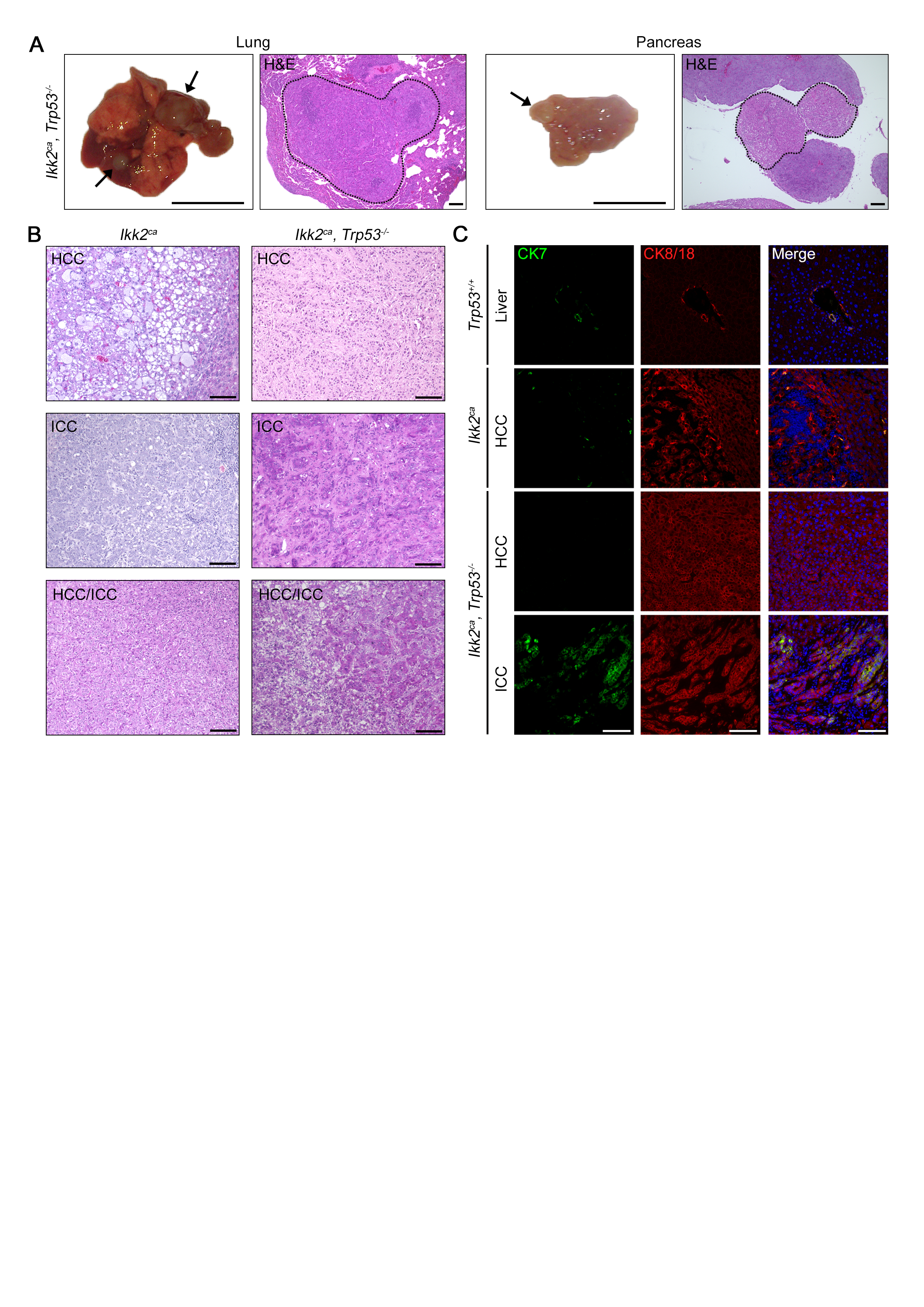


**Figure S3.** Metastasis and tumor differentiation. (Related to Figure 3). (**A**) Macroscopic photographs of metastasis in lung and pancreas of *Ikk2^ca^ Trp53^−/−^* mice (scale bar: 1cm) and corresponding H&E stained microscopic sections (scale bar: 100µm). Arrows point at metastasis. (**B**) Representative photographs of H&E stained liver carcinoma sections from *Ikk2^ca^* and *Ikk2^ca^ Trp53^−/−^* mice of the indicated differentiation (scale bar: 100µm). (**C**) Representative photographs of CK7- and CK8/18-IF as markers for the differentiation of HCC and ICC (scale bar: 100µm).


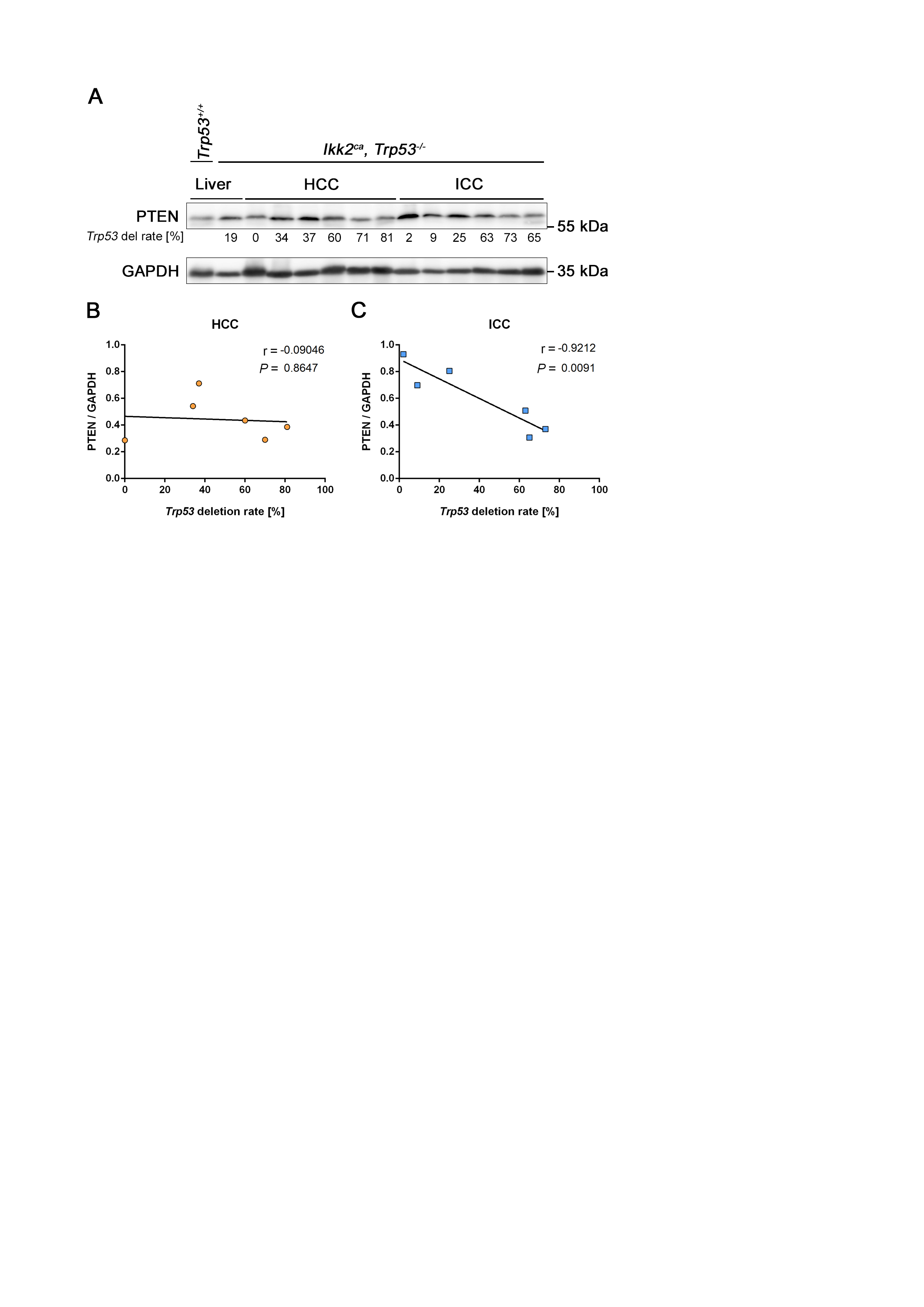


**Figure S4.** Correlation of PTEN expression with *Trp53* deletion status in *Ikk2^ca^ Table* mice. (Related to Figure 4). (**A**) Western blots showing expression of PTEN and GAPDH in whole liver (*Trp53^+/+^*, *n* = 1; *Ikk2^ca^ Trp53^−/−^*, *n* = 1) and tumor lysates from *Ikk2^ca^ Trp53^−/−^* (HCC, *n* = 6; ICC, *n* = 6) mice. The numbers below PTEN blot indicate *Trp53* deletion rate in corresponding tissue of the *Ikk2^ca^ Trp53^−/−^* mice in percentage. (**B,C**) Correlation plots between PTEN protein expression level normalized to GAPDH protein expression and the *Trp53* deletion rate in HCC (**B**; *n* = 6) and ICC (**C**; *n* = 6).


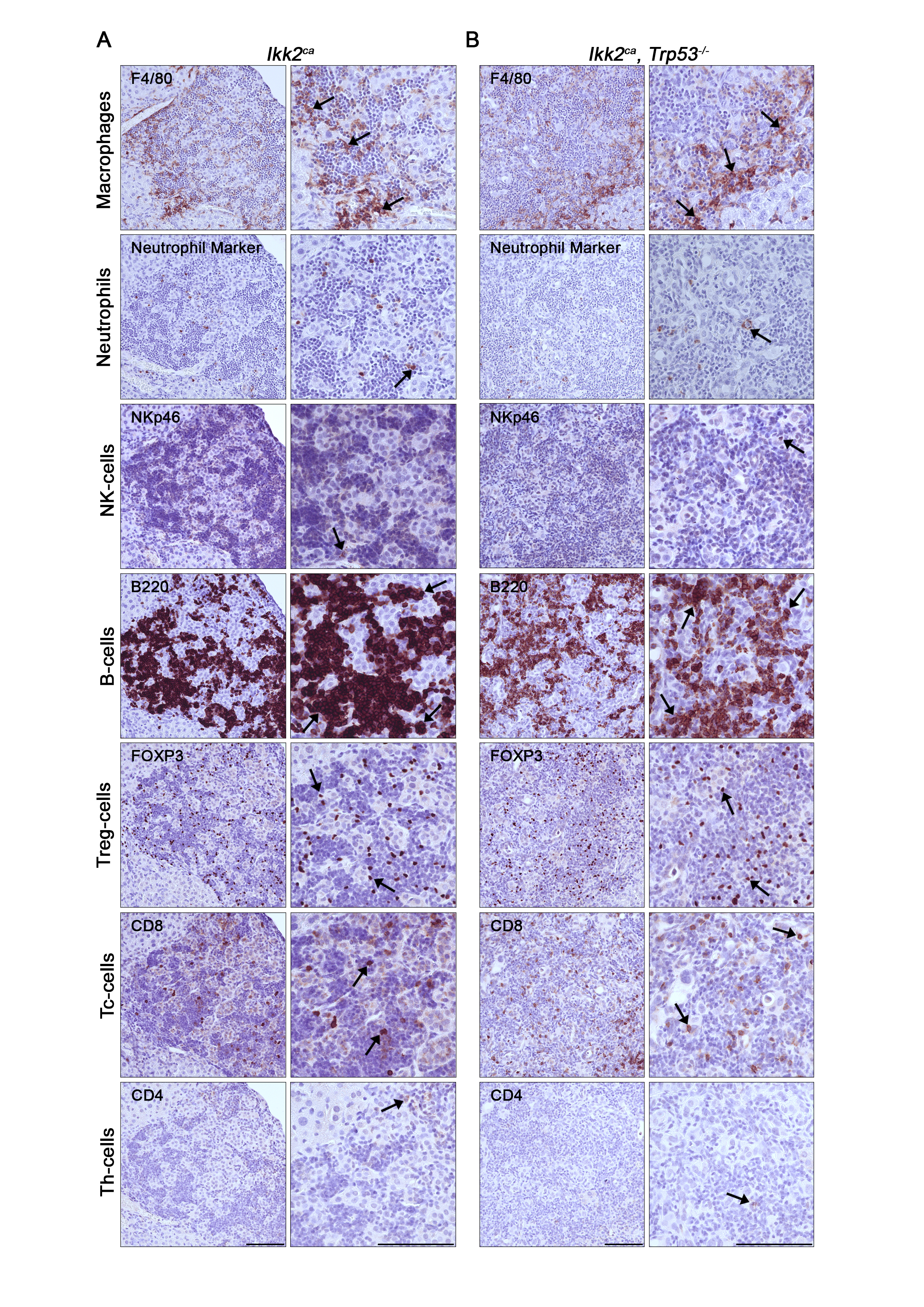


**Figure S5.** IHC of immune cells in ectopic lymphoid structures (ELS). (Related to Figure 5). (**A,B**) High magnification photographs of IHC for the innate immune system: macrophages (F4/80), neutrophils (Neutrophil Marker) and NK cells (NKp46) and the adaptive immune system: B-cells (B220), T regulatory cells (FOXP3), cytotoxic T-cells (Tc-cells, CD8) and T helper-cells (Th-cells, CD4) in ELS of (**A**) *Ikk2^ca^* and (**B**) *Ikk2^ca^ Trp53^−/−^* mice. Left panel images 200×; right panel images 400×, scale bar: 100µm. Arrows point at positive cells.


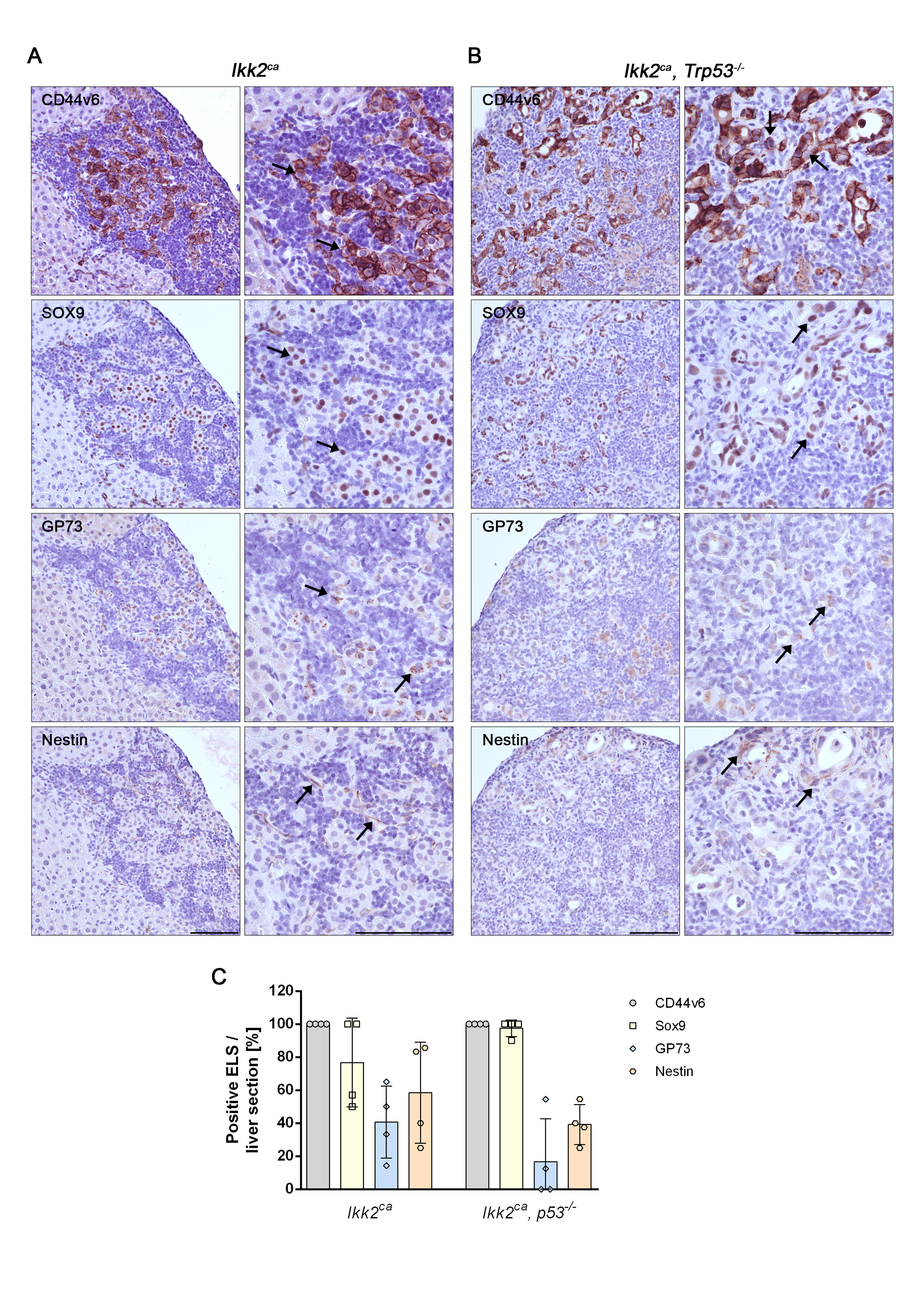


**Figure S6.** IHC of liver progenitor cells in ectopic lymphoid structures (ELS). (Related to Figure 5). (**A,B**) High magnification photographs of IHC for CD44v6, SOX9, GP73 and Nestin in ELS of (**A**) *Ikk2^ca^* and (**B**) *Ikk2^ca^ Trp53^−/−^* mice. Left panel images 200×; right panel images 400×, scale bar: 100µm. Arrows point at positive cells. (**C**) Combined scatter dot plot and bar graph represents the percentage of positive ELS per liver section for the indicated progenitor cell markers in *Ikk2^ca^* (*n* = 4) and *Ikk2^ca^ Trp53^−/−^* (*n* = 4) mice. Mice with more than 4 ELS on the consecutive liver section were analyzed. Data represented as mean ±SD.


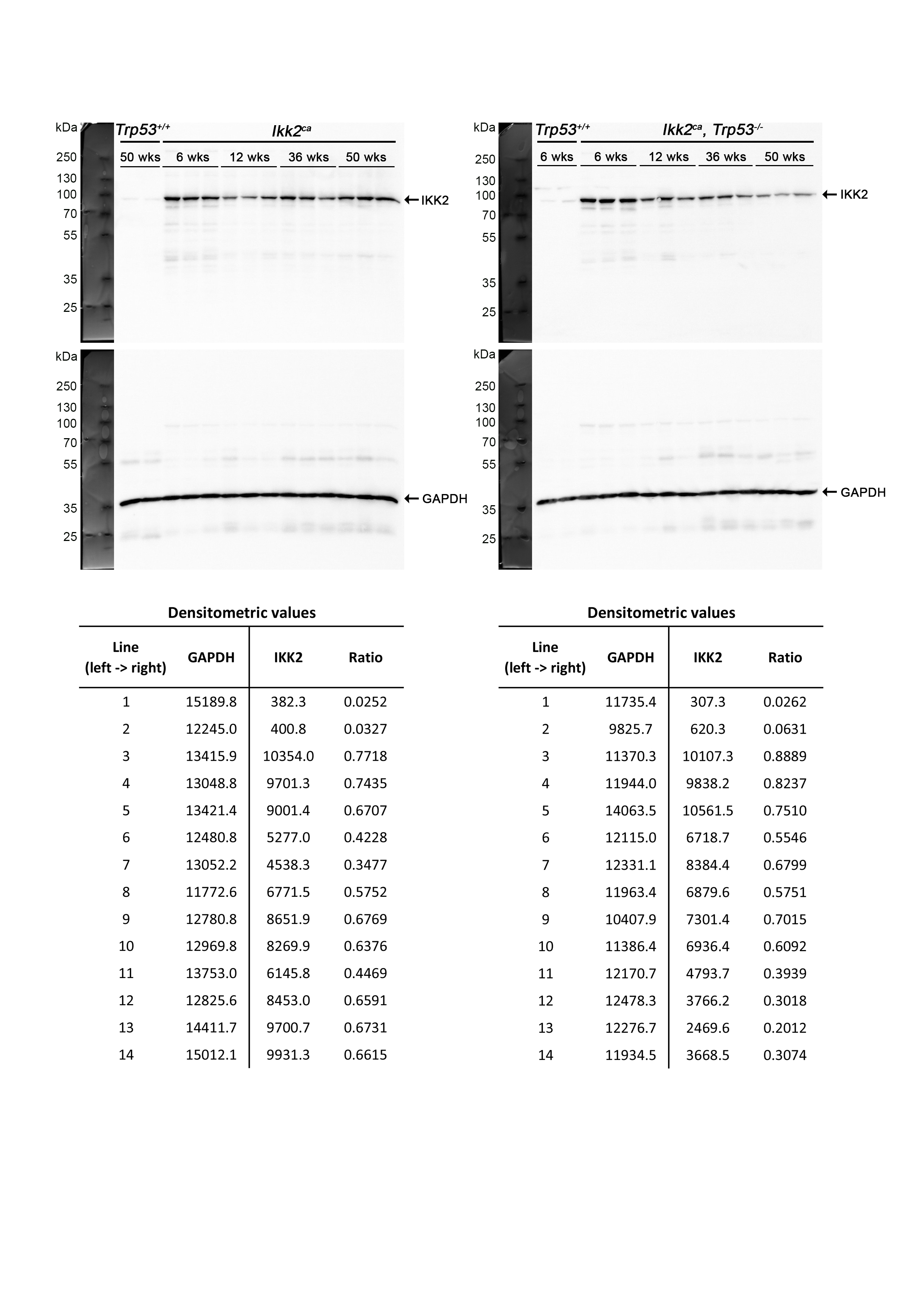


**Figure S7.** Whole length western blots (of western blots shown in Figure 1D), and densitometry readings/intensity ratios.


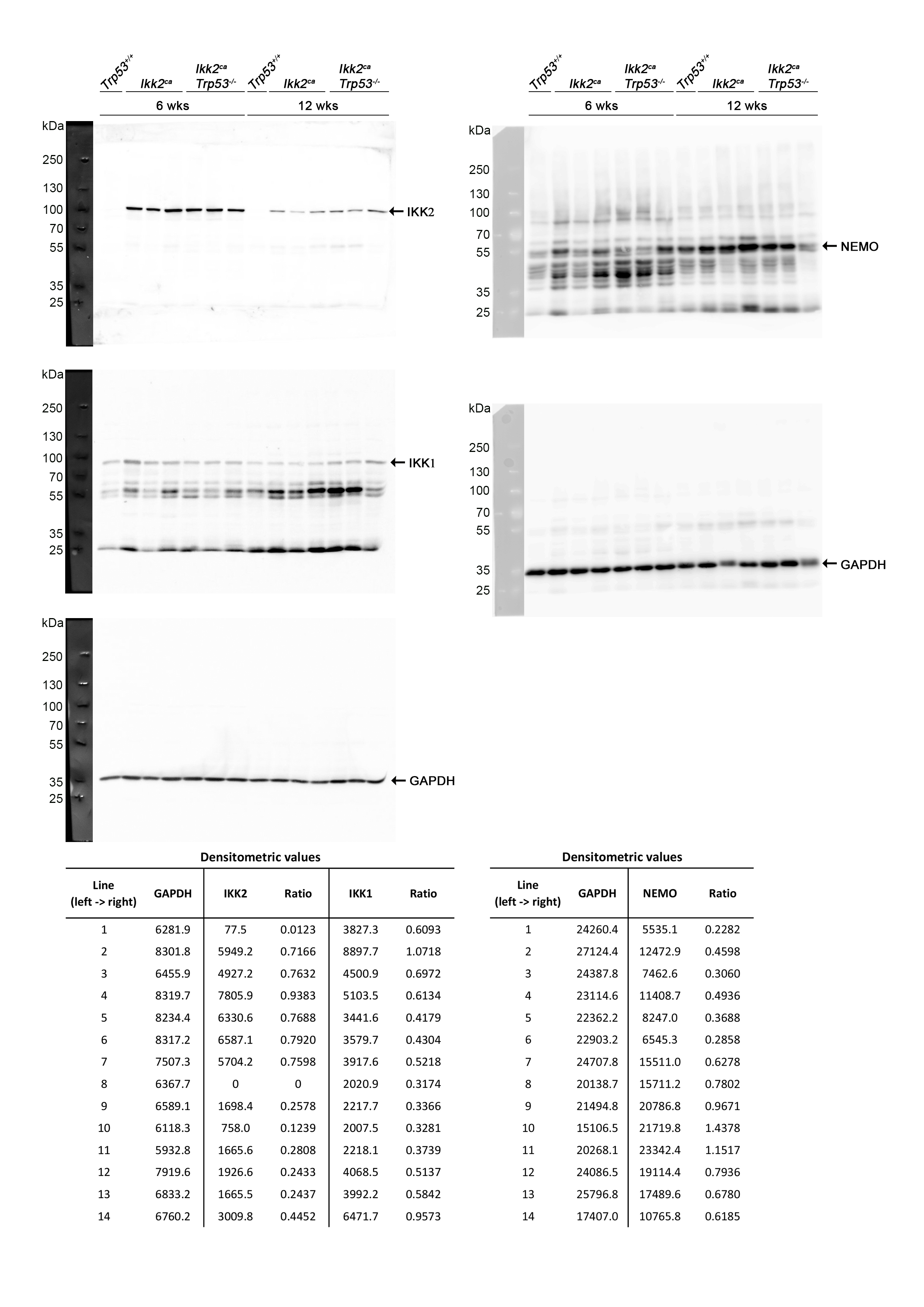


**Figure S8.** Whole length western blots (of western blots shown in Figure S1A), and densitometry readings/intensity ratios.


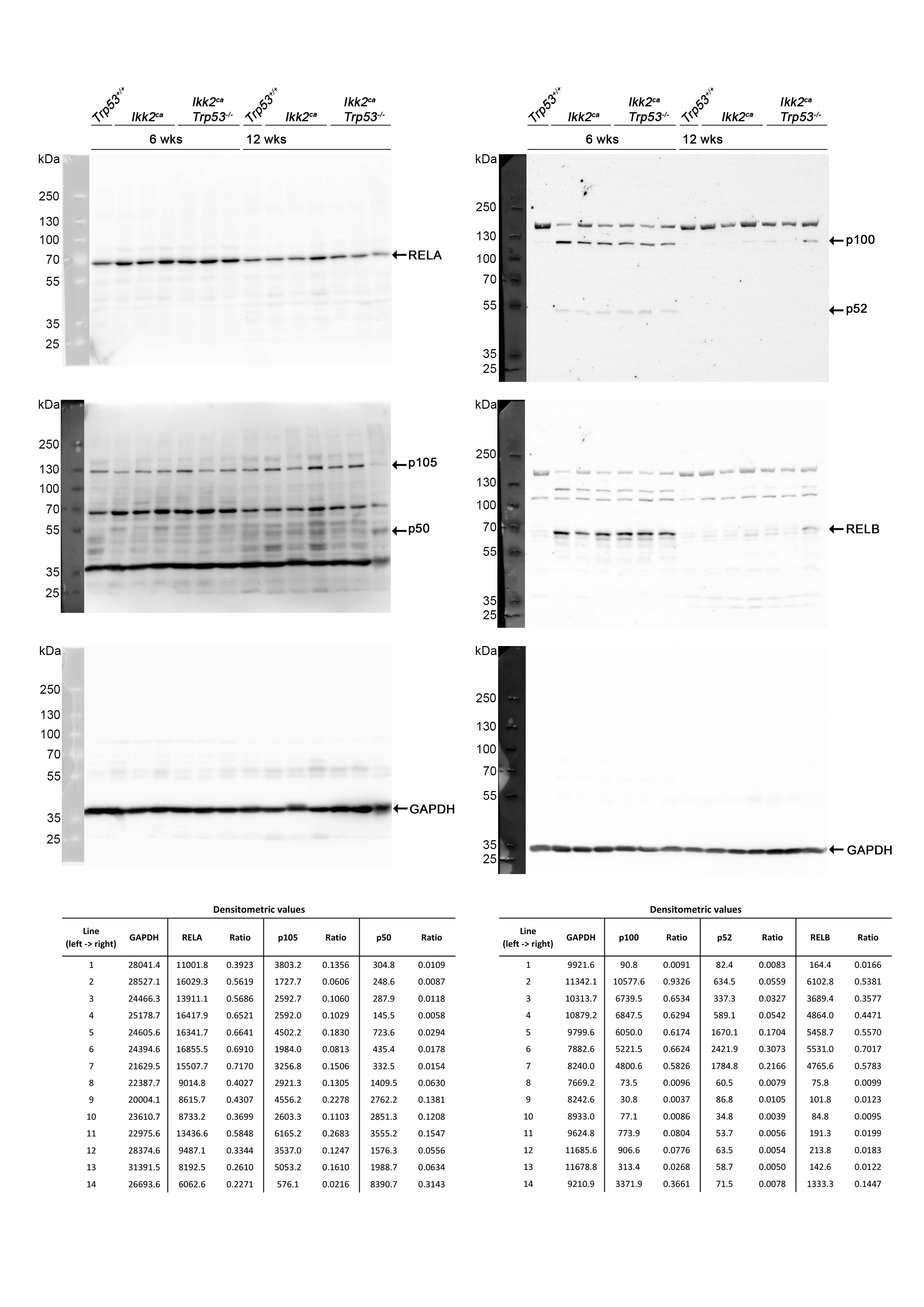


**Figure S9.** Whole length western blots (of western blots shown in Figure S1A, continued), and densitometry readings/intensity ratios.


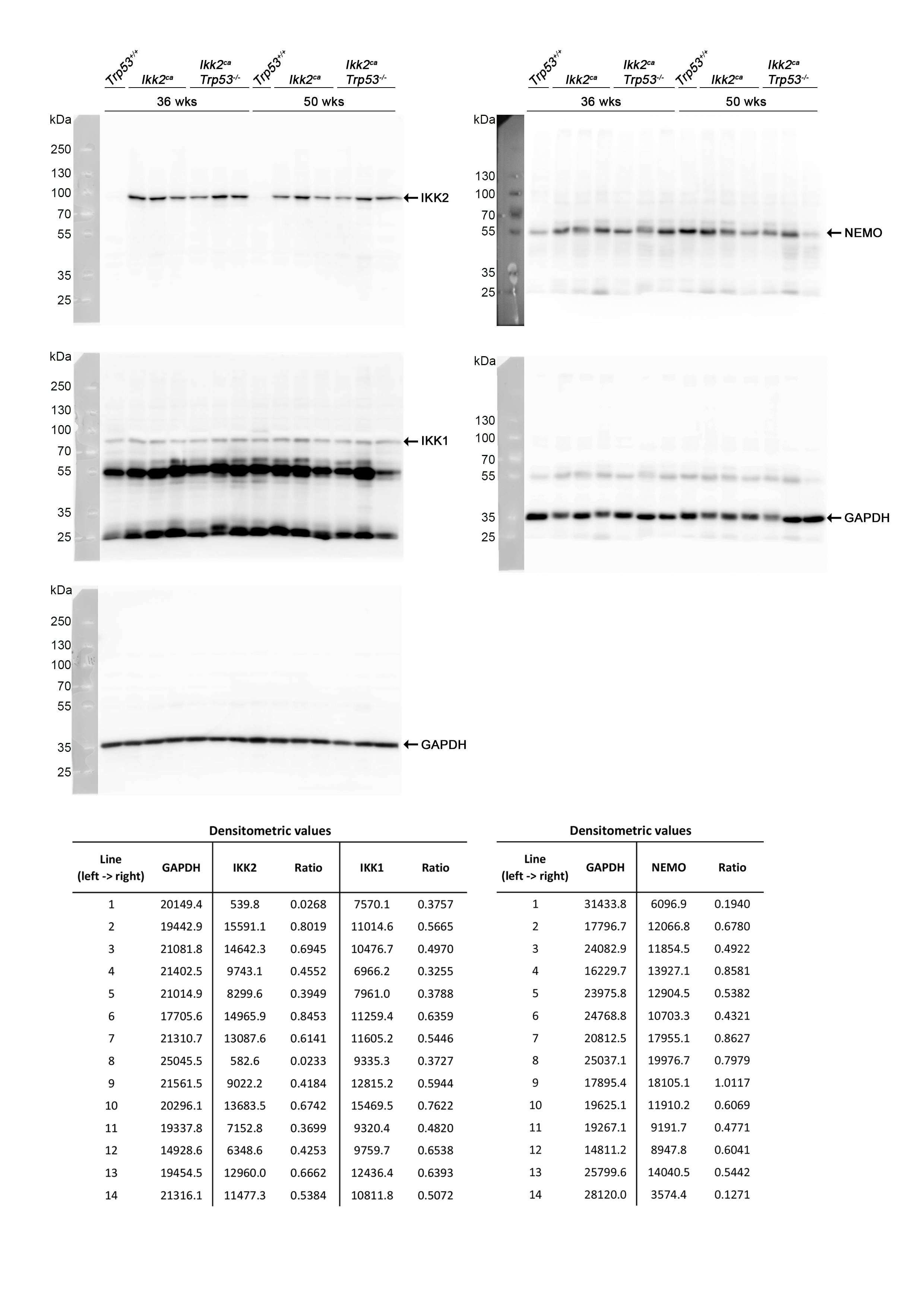


**Figure S10.** Whole length western blots (of western blots shown in Figure S1B), and densitometry readings/intensity ratios.


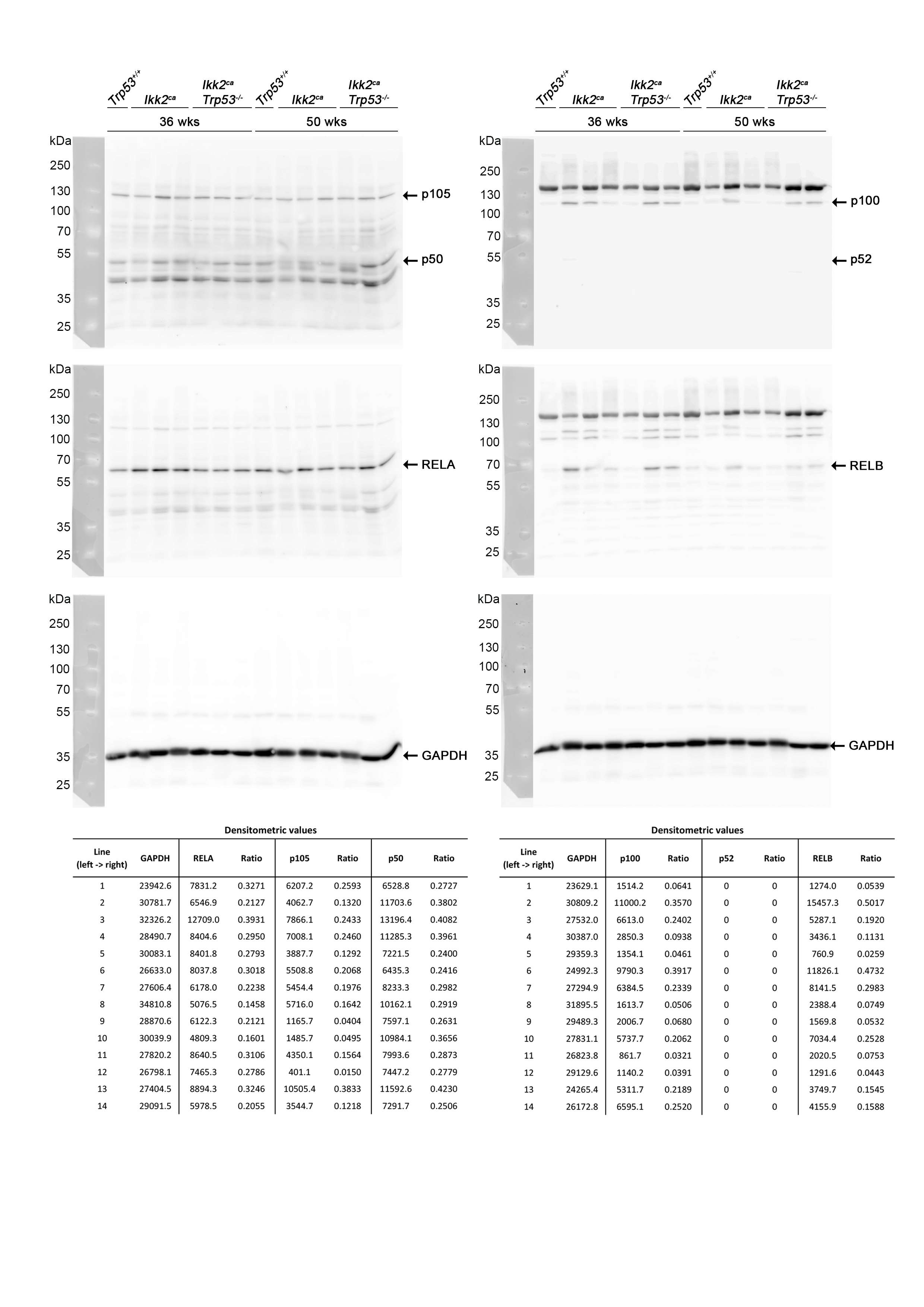


**Figure S11.** Whole length western blots (of western blots shown in Figure S1B, continued), and densitometry readings/intensity ratios.


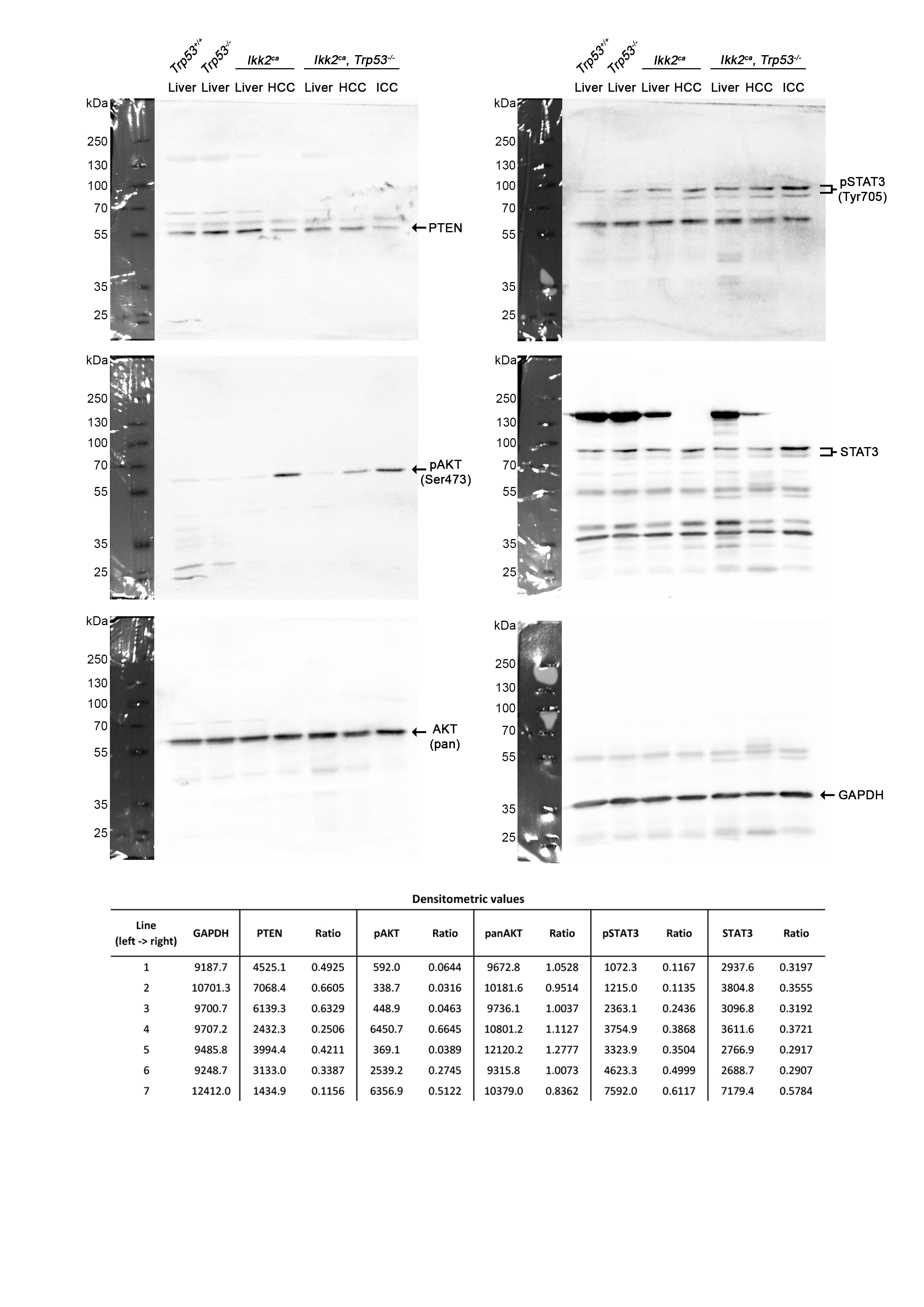


**Figure S12.** Whole length western blot (of western blot shown in Figure 4B), and densitometry readings/intensity ratios.


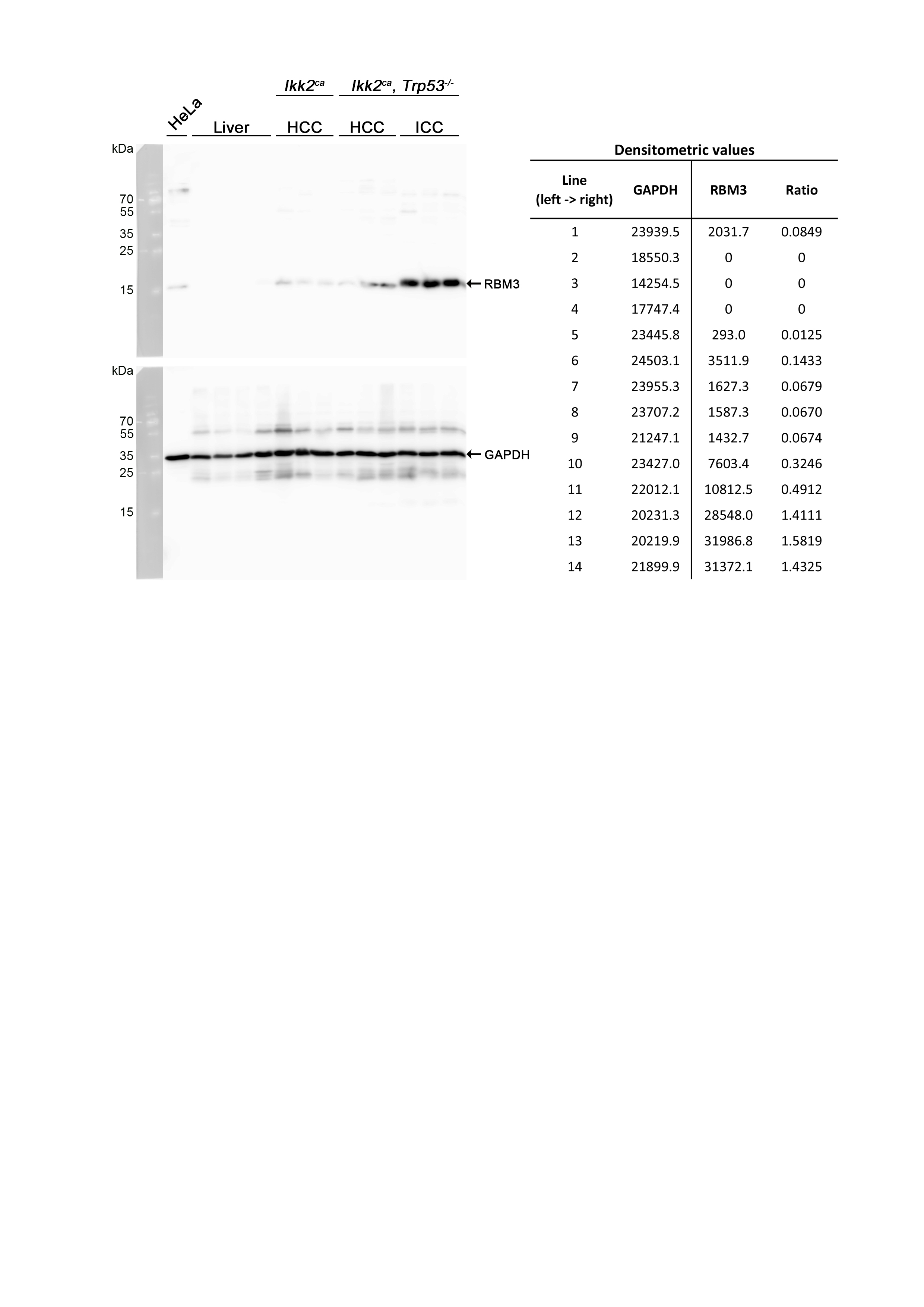


**Figure S13.** Whole length western blot (of western blot shown in Figure 6D), and densitometry readings/intensity ratios.

| 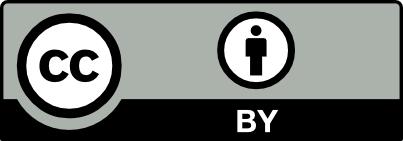 | © 2019 by the authors. Licensee MDPI, Basel, Switzerland. This article is an open access article distributed under the terms and conditions of the Creative Commons Attribution (CC BY) license (http://creativecommons.org/licenses/by/4.0/). |
| --- | --- |
